# Paradigmatic and syntagmatic effects of information status on prosodic prominence – evidence from an interactive web-based production experiment in German

**DOI:** 10.3389/fpsyg.2024.1296933

**Published:** 2024-04-09

**Authors:** Janne Lorenzen, Simon Roessig, Stefan Baumann

**Affiliations:** ^1^IfL-Phonetik, University of Cologne, Cologne, Germany; ^2^Department of Language and Linguistic Science, University of York, York, United Kingdom

**Keywords:** prosody, information status, speech production, prominence, intonation, individual differences

## Abstract

In this paper, we investigate how information status is encoded paradigmatically and syntagmatically via prosodic prominence in German. In addition, we consider individual variability in the production of prominence. To answer our research questions, we collected controlled yet ecologically valid speech by applying an innovative recording paradigm. Participants were asked to perform an interactive reading task in collaboration with an interlocutor remotely via video calls. Results indicate that information status is encoded paradigmatically via the F0 contour, while syntagmatic effects are subtle and depend on the acoustic parameter used. Individual speakers differ primarily in their strength of encoding and secondarily in the type of parameters employed. While the paradigmatic effects we observe are in line with previous findings, our syntagmatic findings support two contradictory ideas, a balancing effect and a radiating effect. Along with the findings at the individual level, this study thus allows for new insights regarding the redundant and relational nature of prosodic prominence.

## 1 Introduction

A crucial goal in communication is to signal discourse meaning via appropriate patterns of *relative prominence* among the words in an utterance. Prominence is a relational property that refers to a speech unit that “stands out” by virtue of a variety of factors pertaining to both meaning and form. Both paradigmatic and syntagmatic aspects have an influence on *prosodic* strength relations, and the aim of the present study is to investigate this interplay in German production data. We can think of the paradigmatic and syntagmatic aspects of prosodic prominence as two axes of the same concept. On the vertical or paradigmatic axis, we consider the prominence of entities occurring in the same phrasal position but encoding different discourse meanings. This axis takes into account prominence relations *across* different utterances. On the horizontal or syntagmatic axis, we consider the prominence of multiple successive entities. This axis hence accounts for prominence relations *within* a phrase or utterance. The layer of *meaning* we are looking at is the information status of referents, i.e., their level of givenness in discourse. The *form* relates to both phonetic and phonological features of prosodic analysis, namely gradual cues such as F0 height and excursion, duration or periodic energy, as well as categorical distinctions between pitch accent type and status (i.e., prenuclear vs. nuclear).

**Figure 1:**
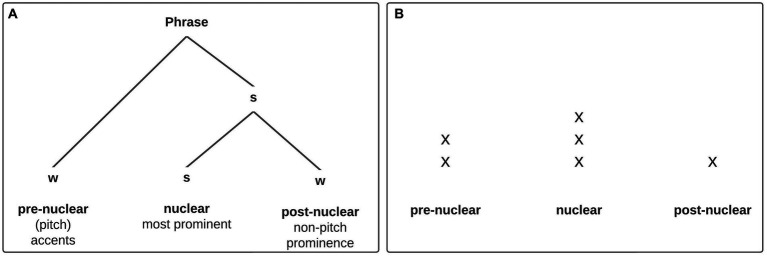
**(A)** Basic (ip-)phrase level metrical structure (Calhoun, 2010, p. 4); prenuclear and nuclear prominences are pitch accents, i.e., show tonal movement, while postnuclear prominences are non-tonal, especially durational. **(B)** The same structure presented as a metrical grid to illustrate the prominence relation between prenuclear and postnuclear accents more adequately.

In our formal analysis we follow the ‘metrical branch’ of prosodic phonology, proposed, for example, by Ladd (2008, 2014), and broadly defined as the hierarchical structure of utterances and their syntagmatic strength relations. A crucial insight is that there is no unified set of suprasegmental features and domains emerging from the metrical perspective (Ladd, 2014, pp. 50, 74). Nevertheless, there is a selection of phonological constituents which are considered relevant for metrical representations, organized in the *prosodic hierarchy* (e.g., syllable, foot, phonological word, intermediate phrase, intonational phrase, utterance). The tonal structure of the utterance adds another layer to this hierarchy. There are two types of tones associated with metrically important positions in the prosody hierarchy, which prototypically fulfill two different functions: boundary tones mark edges and are associated with higher-level phrases [e.g., H- indicates the end of an intermediate phrase (ip), H% marks the end of an intonational phrase (IP)], while pitch accents (starred elements, e.g., H*) are associated with prominent syllables and mark strong positions, or heads, in larger phrases. Figure 1A shows the abstract strength relation among prominent elements at the intermediate phrase level in a metrical tree, indicating that the nuclear accent is most prominent, and also structurally most important (only dominated by s-nodes; see, e.g., Calhoun, 2010). Pre-and postnuclear accents are secondary in relation to the nucleus, which is the only obligatory accent in an intermediate phrase. However, while the metrical tree adequately depicts the structural relations within the phrase (i.e., the postnuclear element is more closely tied to the nuclear element than prenuclear constituents would be) it does not mirror the actual prominence relation between pre-and postnuclear accents: the prenuclear element is only dominated by a w-node whereas the postnuclear element is dominated by an s-node at a higher level, although prenuclear accents are generally assumed to be *more* prominent than postnuclear accents (see Ladd, 2008, pp. 262–263; Calhoun, 2010, p. 3). In fact, this (empirically more reasonable) relation can better be captured in a metrical grid, where prominence is assigned via the number of beats on units within an utterance (see Figure 1B, e.g., Hayes, 1995).

Although both paradigmatic effects of meaning-related factors and syntagmatic effects of (form-related) prosodic structure on prominence have been addressed independently in previous studies many times before, they have rarely been dealt with in conjunction. In this paper, we attempt to take a more comprehensive look at the (prosodic) prominence relations between two referents in a sentence and their influencing factors – broadened by a close investigation of speaker-specific differences.

### 1.1 Prosodic marking of information status in West Germanic (the paradigmatic perspective)

**Figure 2:**
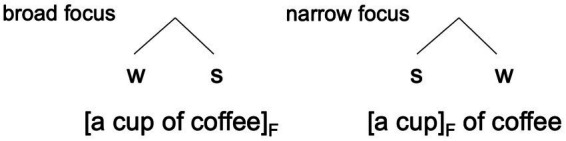
Metrical representation of information structural oppositions (Ladd, 2008, p. 271).

Metrical strength relations do not only depend on prosodic aspects of an utterance, but often reflect meaning-related choices, i.e., the semantic-pragmatic and syntactic properties of the utterance that are related to the previous discourse. For example, a difference in information structure (or rather its prosodic marking) can be expressed by a different mapping of information structural domains onto metrical structure (see Ladd, 2008; Calhoun, 2010), as in Figure 2. Here, the difference between the broad focus structure of the phrase *a cup of coffee* (Figure 2, left), which would be appropriate as an answer to the question *‘What would you like to drink?’*, and the narrow (or contrastive) focus structure (Figure 2, right), being valid in a context such as *‘I’d rather like a pot of coffee and not…’*, can be represented in an efficient and elegant fashion simply by reversing the weak and strong nodes. The minimalist tree is a shorthand for the fact that in broad focus, *coffee* occupies the strongest position (realized by the nuclear accent), whereas in narrow focus, the nuclear accent moves to *cup* (and *coffee* is deaccented). If more complex structures containing this phrase are built (e.g., *five francs seventy-five centimes and a cup of pretty tasteless coffee*; Ladd, 2008, p. 272), the w-s relation between *cup* and *coffee* still signals broad focus and s-w signals narrow focus. This representation of prosodic strength relations is arguably very coarse-grained. There may be meaningful gradient variation in prosody, which is not captured in such models (e.g., Ladd, 2022; Roessig, 2024).

In the present study, we are concerned with information status, which can be regarded as another layer of information structure alongside focus. Following Chafe (1994), information status can be defined as the degree of cognitive activation or givenness of a referent in the discourse. It can be determined based on whether a referent has previously been established (often explicitly mentioned) in the current discourse and is thus already “active,” e.g., the second occurrence of *bus* in (1), or has been newly introduced from a previously “inactive” state, e.g., *a bus* in (1) and (2). Inactive referents are classified as *new*, active ones as *given.* A third category consists of *accessible* referents, which have not been explicitly mentioned but are retrievable through the context and thus are considered to be cognitively “semi-active,” e.g., *the driver* in (2).

(1) I got on a bus (*new*) yesterday and the bus (*given*) was crowded.

(2) I got on a bus (*new*) yesterday and the driver (*accessible*) was drunk.

(examples adapted from Prince, 1981)

Information status has been linked to various, quite diverse concepts such as (shared) knowledge, consciousness, predictability as well as (un-)importance (see Prince, 1981; Baumann and Riester, 2012, for overviews). For the purpose of the present study, we will adopt Chafe’s idea of the information status of a referent or concept as part of the common ground between speaker and listener, which is more or less transparently derivable from the previous discourse context. For the sake of simplicity, we will restrict ourselves to Chafe’s three-way distinction of *new-accessible-given*, although more refined schematizations of information status are available, such as the *RefLex scheme*, which distinguishes a lexical and a referential level of givenness (Baumann and Riester, 2012, 2013; Riester and Baumann, 2017). In our study, we focus on the contrast between *new* and *accessible* referents.

Different levels of information status are marked by prosody in German and other West Germanic languages, in particular via differences in prosodic prominence. Discourse-*given* referents are typically produced least prominently, discourse-*new* referents are prosodically most prominent and *accessible* referents occupy an intermediate position on the prosodic prominence scale (for German: Féry and Kügler, 2008; Baumann and Riester, 2013; for English: Ito et al., 2004; Chodroff and Cole, 2019; for Dutch: Swerts et al., 2002). Earlier accounts have focused on a binary distinction between *new* and *given* referents in terms of accentuation or deaccentuation, respectively (Halliday, 1967; Cruttenden, 2006; Ladd, 2008). However, studies have shown that there is no one-to-one relation between deaccentuation and givenness but rather a probabilistic mapping (Terken and Hirschberg, 1994; Calhoun, 2010). Furthermore, like *new* referents, *accessible* referents are typically accented. Nevertheless, referents can be distinguished according to their information status via more fine-grained prosodic categories such as pitch accent type or gradient phonetic parameters such as F0 alignment and scaling, duration and intensity: *New* referents are typically produced with more high and rising accent types (e.g., Pierrehumbert and Hirschberg, 1990; Baumann and Riester, 2013) and with more extensive F0 excursion, later F0 peak alignment, longer duration and higher intensity when compared to *given* or *accessible* referents (e.g., Féry and Kügler, 2008).

Aylett and Turk (2004), drawing on information theory (*cf.* Shannon, 1948), proposed a principled account of the inverse relationship between ‘linguistic redundancy’ and ‘acoustic redundancy’ (i.e., prosodic prominence), known as the *Smooth Signal Redundancy Hypothesis* (SSRH). According to the SSRH, information is distributed evenly across the speech signal: Linguistically highly redundant information, which is already easily retrievable or predictable for the listener, is prosodically attenuated (i.e., acoustically less redundant or less prominent), while linguistically less redundant, harder-to-retrieve information is prosodically highlighted making it acoustically more salient (claimed at least for West Germanic languages). This yields a “smooth redundancy profile” in speech, ensuring robust communication, while at the same time reducing articulatory effort. The notion of predictability, which is central to the definition of linguistic redundancy, is directly related to information status, in that *given* referents can be regarded as highly predictable from the discourse context, *accessible* referents as somewhat predictable and *new* referents as unpredictable.

The original study conducted by Aylett and Turk (2004) confirmed this relation by showing that duration depends on word frequency, syllabic trigram probability and reference mention in English. This finding has since been extended, for example, to the relation between fundamental frequency modulations and predictability as captured by semantic focus and utterance probability (Turnbull, 2017). The Smooth Signal Redundancy Hypothesis thus systematically accounts for the paradigmatic relation observed between information status and prosodic prominence.

### 1.2 Prominence relations and the tonal context (the syntagmatic perspective)

It is not only the information status depending on the discourse context – and, as a consequence, the paradigmatic choices at the lexical and syntactic level – that influences an utterance’s metrical structure, but also the *syntagmatic prosodic context* within an utterance. It is well known, for example, that the prominence of a word or syllable can be enhanced by reducing the pitch of neighboring syllables (Gussenhoven et al., 1997), which is exploited, for example, in focus marking in the form of post-focal compression (e.g., Xu et al., 2004). Furthermore, the relative pitch height of two accents and their distance affect the perceived degree of prominence. Schettino and Wagner (2015) showed that in a German corpus of read speech, which has been annotated for prominence levels (Bonn Prosodic Database; Heuft, 1999), a pitch accent was judged as *more* prominent the further away it was from a previous accent. At the same time, speakers produced these more distant accents proportionally somewhat less steeply and less delayed – and thus *less* prominently. This result not only confirms that we are dealing with both relative and cohesive patterns of prominence, but it is also in line with the declination effect (see Pierrehumbert, 1979), indicating that a lower peak accent following a higher one will be perceived as just as high and prominent as the preceding accent. This effect is even stronger the later the second accent occurs in an utterance.

**Figure 3:**
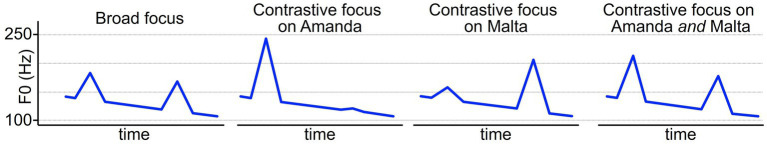
Target sentence and prototypical pitch contours for different focus conditions produced on the utterance *A’manda gaat naar ‘Malta* “Amanda is going to Malta,” adapted from Rump and Collier (1996, p. 9).

Along similar lines, Rump and Collier (1996) investigated the effects of focus type on perceived appropriateness of pitch peak height in two referents within the same utterance. As expected, higher peaks were judged more appropriate by Dutch listeners for two subsequent referents that were both produced in contrastive focus than for two referents in broad focus (see Figure 3). Interestingly, however, when a single referent in contrastive focus was preceded or followed by a referent in the background, higher peaks were found to be more appropriate for the contrastive referent than when it was preceded or followed by another referent in contrastive focus. Similarly, Bishop (2017) found that in English SVO sentences, listeners dispreferred a prenuclear accent on the verb when the subsequent object was in narrow focus, but they showed no preference when it was in broad focus. These findings suggest that information structure is not only encoded locally (i.e., by position and type of the nuclear pitch accent) but that it is distributed across the sentence in the *prominence relation* of multiple accents.

In line with these observations, Roessig (2024) in a production study on German found an inverse relation of prosodic prominence between prenuclear and nuclear constituents depending on focus type. Prenuclear words occurring before nuclear words in corrective focus were realized less prosodically prominent (in terms of F0 height and syllable duration) than prenuclear words before words in (non-corrective) narrow focus and least prominently in a broad focus domain, while nuclear words in corrective focus were realized more prominently than nuclear words in narrow focus, which were in turn more prominent than nuclear words in broad focus. In another production study on German, Kügler and Gollrad (2015) also found that a prenuclear accent occurring before a nuclear accent in broad focus was realized with a higher peak F0 than a prenuclear accent occurring before a nuclear accent in contrastive focus. In a follow-up perception experiment they confirmed that listeners were more likely to interpret an utterance as conveying contrastive information when the F0 peak of the nuclear accent was higher than that of a prenuclear accent compared to when the F0 peak of the nuclear accent was lower than the prenuclear accent’s. Information structure thus appears to be encoded in the *balancing* of the prenuclear and nuclear accents.

In contrast to these findings, Gussenhoven and Rietveld (1988) observed a positive correlation between the perceived prominence of two subsequent pitch peaks in Dutch listeners. In their prominence rating task, participants rated the prominence of a second peak lower if a preceding peak was realized with a lower F0 and thus less prominently, while a higher F0 on the first peak increased the prominence rating of a following peak. Higher prosodic prominence of the initial peak thus led to a perceived increase of prominence on a second peak irrespective of its realization. This suggests that prominence has a *radiating* effect, spreading onto following entities. This somewhat unexpected observation has become known as the *Gussenhoven-Rietveld Effect*. Gussenhoven and Rietveld (1988, p. 366) argue that the effect is potentially driven by the expectations of the listeners: If they encounter a low initial peak, they expect to hear another low peak next, which attenuates the perceived prominence of the actual peak that follows. At the same time, the study revealed the opposite effect for intensity, in that lower intensity of the first accented syllable increased the perceived prominence of the second one. Here, they argue that loudness is evaluated directly relative to the signal, i.e., lowering the intensity in one part of the signal leads to a perceived increase in loudness in the remaining parts. This observation seems to be more in line with the findings by Rump and Collier (1996) and Roessig (2024).

Ladd et al. (1994) partially replicated the Gussenhoven-Rietveld Effect with English listeners, but only in phonetically untrained participants and on a stimulus set containing a slightly lower second pitch peak (140 Hz). In a stimulus set containing a higher second peak (160 Hz), the opposite effect emerged in that an increase in the pitch of the first peak led to a decrease of perceived prominence on the second peak [akin to the balancing effects observed, for example, by Rump and Collier (1996) and Roessig (2024)]. This finding was confirmed in a second experiment with more values for the second pitch peak. According to Ladd et al. (1994), the Gussenhoven-Rietveld Effect replicated only in the condition with a lower pitch on the second peak since such a contour reflects what listeners consider a “normal” pitch range. A higher pitch range, on the other hand, implies an emphatic realization, which leads to the opposite effect. They concluded that while prominence is evaluated in a global fashion in non-emphatic productions, emphasis as a paralinguistic cue may override this interpretation (Ladd et al., 1994, p. 98).

To summarize, while there is evidence that prosodic prominence is influenced by the context at a syntagmatic level, it is not clear how such effects would materialize in the production of different levels of information status in German. In the case of a balancing or trade-off effect akin to the findings by Rump and Collier (1996) for the single contrastive focus conditions, Kügler and Gollrad (2015), Bishop (2017), and Roessig (2024), an utterance could be expected to have a fixed prominence budget, which is distributed across the referents within an utterance according to their information status. For example, a *new* referent following or preceding an *accessible* referent would be more prominent than a *new* referent following or preceding another *new* referent. A radiating effect in production, analogous to the perceptual effect observed by Gussenhoven and Rietveld (1988) and Ladd et al. (1994), would imply that a more prominent referent in the utterance would also raise the prominence of the other referent, e.g., an *accessible* referent followed or preceded by a *new* referent would be more prominent than an *accessible* referent followed or preceded by another *accessible* referent. The contradictory findings in previous studies inspire different expectations for our present study, which calls for an exploratory approach.

### 1.3 Speaker-specific variability (the individual perspective)

Prosodic prominence is multidimensional, encompassing a variety of cues related to timing, changes in F0 and spectral characteristics of the speech signal (e.g., Fry, 1955; Sluijter and Van Heuven, 1996; Kochanski et al., 2005; Baumann and Winter, 2018; Roessig et al., 2022). Prosodically prominent entities are produced with steeper and more rising F0 contours, longer durations and higher intensity than less prominent entities in West Germanic languages. Due to the inherently redundant nature of prosodic prominence, pragmatic categories differentiated via prominence levels are thus encoded by a set of different cues producing the same effect. This redundancy may consequently enable a higher degree of individual variability, especially in the choice of prominence cues. Instead of encoding contrasts maximally redundantly by exploiting all prosodic cues to prominence, speakers may focus their production efforts only on specific cues.

Inter-individual variability is ubiquitous in speech production and perception and has recently received much attention in prosodic research. Previous studies have found, for example, considerable inter-individual variability in focus type marking. For instance, German speakers differed in the type and number of prosodic cues they employed to distinguish between broad, narrow and contrastive focus (Cangemi et al., 2015). Some speakers encoded a three-way contrast via multiple prosodic cues, e.g., peak alignment, peak height and word duration, while other speakers used only single cues to differentiate between two focus types, e.g., word duration to distinguish broad from narrow and contrastive focus and the presence or absence of prenuclear accents to distinguish broad and narrow focus from contrastive focus. These differences in the robustness of focus type encoding have implications for the interpretation of the utterances making some speakers more or less intelligible to the listener.

Similarly, Ouyang and Kaiser (2015) observed that American English-speaking individuals differed in how strongly they encode informativity (i.e., focus type, contextual probability, and word frequency) via the F0 contour, in that some speakers made larger differences in terms of F0 excursion between the levels of these three variables. Kim (2019) found considerable individual differences in prosodic cues to phrase boundaries in American English speakers. While all speakers produced pauses at IP boundaries, they differed in pause durations. In addition, most but not all speakers employed pitch reset at IP boundaries and there was substantial variability in the scope of phrase-final lengthening across speakers.

Concomitantly, perception studies have observed substantial variability in the cues listeners attend to in the decoding of prosodic prominence (Cangemi et al., 2015; Baumann and Winter, 2018). Baumann and Winter (2018) in a study on German, for example, identified two groups of listeners, the larger group (about two thirds of the sample) attending primarily to cues related to the F0 contour, while the smaller group seemed to rely on non-prosodic cues such as word frequency, part-of-speech and argument structure in addition to duration. Cangemi et al. (2015) found different levels of proficiency in German listeners’ ability to distinguish between focus types. What is more, specific listeners seemed to be particularly adept at interpreting focus types as produced by specific speakers but were less reliable in interpreting the productions of other speakers. In order to arrive at a comprehensive understanding of prominence production, we thus need to consider individual variability in our investigation.

### 1.4 Research questions and expectations

In this paper, we address three main research questions (RQs) related to paradigmatic, syntagmatic and individual aspects of the prosodic encoding of information status:

RQ1: How and to what extent is the information status of two successive referents encoded by prosodic prominence (at a paradigmatic level)?

RQ2: How and to what extent does the information status of two successive referents affect the prosodic prominence relation between them (at a syntagmatic level)?

RQ3: Do individual speakers use different strategies in their encoding of information status and if so, how are these strategies characterized?

Based on previous research, we derive the following expectations:

(RQ1) Paradigmatically, *new* referents should be produced with higher prosodic prominence than *accessible* referents, both at the phonological and the phonetic level, e.g., by a larger number of rising accent types, with more extensive F0 changes, longer duration and followed by more phrase boundaries.

(RQ2) Previous studies suggest two potential outcomes concerning syntagmatic effects on prominence relations: a balancing effect or a radiating effect. Given that there seems to be more (recent) evidence in favor of the balancing effect, we assume this to be the more likely outcome, which we will thus take as a basis for the comparisons of the posterior estimates presented in Section 3.2.

(RQ3) We expect speakers to differ in the type of prosodic cues they employ as well as the strength of the encoding of the *new-accessible* contrast. Due to the morpho-syntactic marking of (in-) definiteness of each referent by the preceding article as a cue to information status, prosody may even be regarded as a redundant cue by some speakers.

## 2 Materials and methods

### 2.1 Reading material

**Figure 4:**
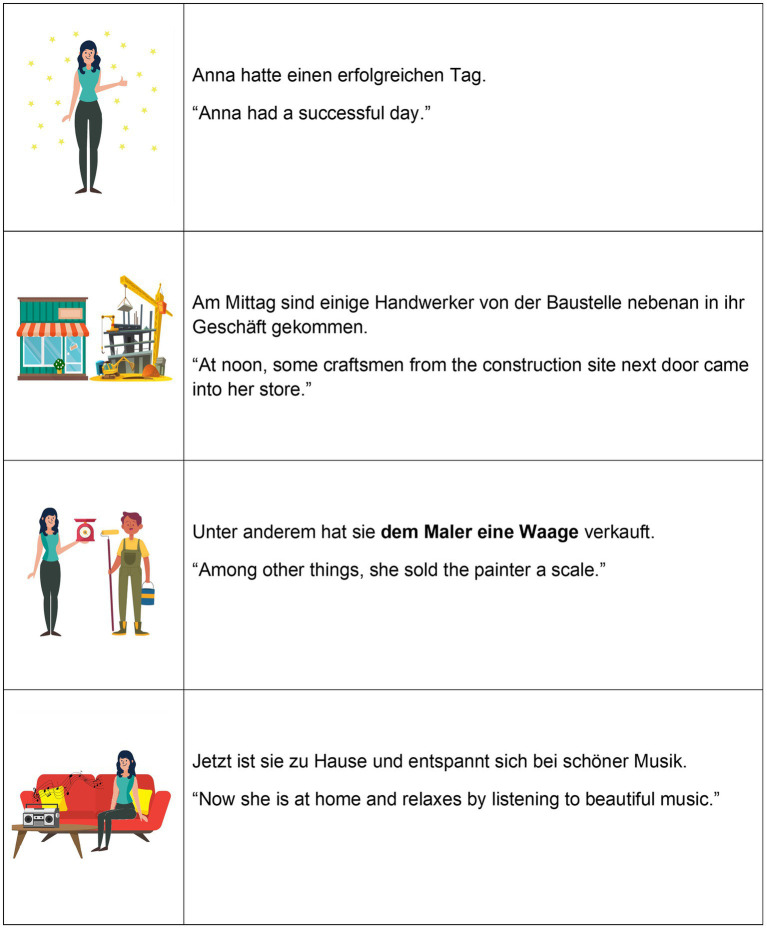
Example story and corresponding pictures with an *accessible* direct object (the painter) and a *new* indirect object (a scale), *[accessible, new]* condition.

To address our research questions, we collected data from a reading task. The analysis was based on 16 disyllabic target words with stress on the initial syllable, which were embedded in eight different short stories consisting of four sentences each (see Figure 4 for an example). Crucially, the target sentence, i.e., the third sentence in the story, included two consecutive target words in indirect and direct object roles. The indirect objects (Word1) always referred to people by their professions or, in one case, a family relation, e.g., *Lehrer* (“teacher”) or *Nonne* (“nun”). The direct objects (Word2) were everyday items, e.g., *Geige* (“violin”) or *Säge* (“saw,” see Appendix for full list of target words and stories). Target words belonged to comparable frequency classes according to the *Wortschatz Leipzig* corpus.^1^ We also ensured that the target words consisted of mostly sonorous segments to facilitate the analysis of the F0 contour.

The target words were either referentially and lexically *new* or *accessible* through the context provided in the previous sentence (in the example story in Figure 4, *the painter* is cognitively activated to some extent, i.e., *accessible,* by the scenario of a construction site mentioned before, while *a scale* is cognitively inactive, i.e., *new*).^2^ *New* referents were preceded by an indefinite article, *accessible* referents by a definite article. We chose not to include *given* referents as they are often deaccented, which would pose difficulties for the comparisons of some parameters to accented referents. Each of the eight stories was devised in all four possible combinations of *new* and *accessible* target words (*[new, new]*, *[new, accessible]*, *[accessible, new]*, *[accessible, accessible]*), but participants saw only one version of each story in a Latin square design. This served to prevent target words from becoming lexically *given* through repeated mentions. Each participant produced two stories for every possible information status combination. Target utterances were always produced in broad focus, either with a prenuclear or nuclear pitch accent on the target word.

### 2.2 Visual material

Speakers saw four pictures, each corresponding to one sentence of the story presented at the same time as the reading material. The pictures were also used in a staged picture sorting task (see Section 2.3 for more details). Pictures were created using resources from Freepik.com.^3^ The first and last picture usually showed the protagonist of the story in different settings (in one case, the protagonist did not occur before the second picture, while the first picture showed a landscape). The second picture illustrated the setting of the main plot of the story (sometimes including the protagonist), which was related to one or both target words if they were *accessible* or unrelated if they were *new*. The third picture portrayed an interaction between the protagonist and another human interlocutor (the direct object, Word1) involving an item of interest (the indirect object, Word2). In this picture, both target words were displayed, which rendered them visually *given* for the speaker. The speaker was informed that the listener did not see the pictures until after the story was read aloud, so that the visual givenness was not common ground. As expected, we did not observe any deaccentuation of the target words, which would have been a strong indication of the referents being interpreted as *given* by the speaker. In addition, visual priming is known to only play a subordinate role in referent activation (Baumann and Hadelich, 2003).

### 2.3 Participants and data collection

We recorded 32 native speakers of German (8 identified as male, 24 as female, aged 20 to 38 years). Participants originated from seven different federal states of Germany and spoke no clearly detectable dialect. Two participants grew up bilingually with Russian or French as a second language but reported German to be their dominant language. Most were students at the time of recordings (*n* = 27). Participants were volunteers and received no compensation. They provided their written informed consent to participate in this study.

**Figure 5:**
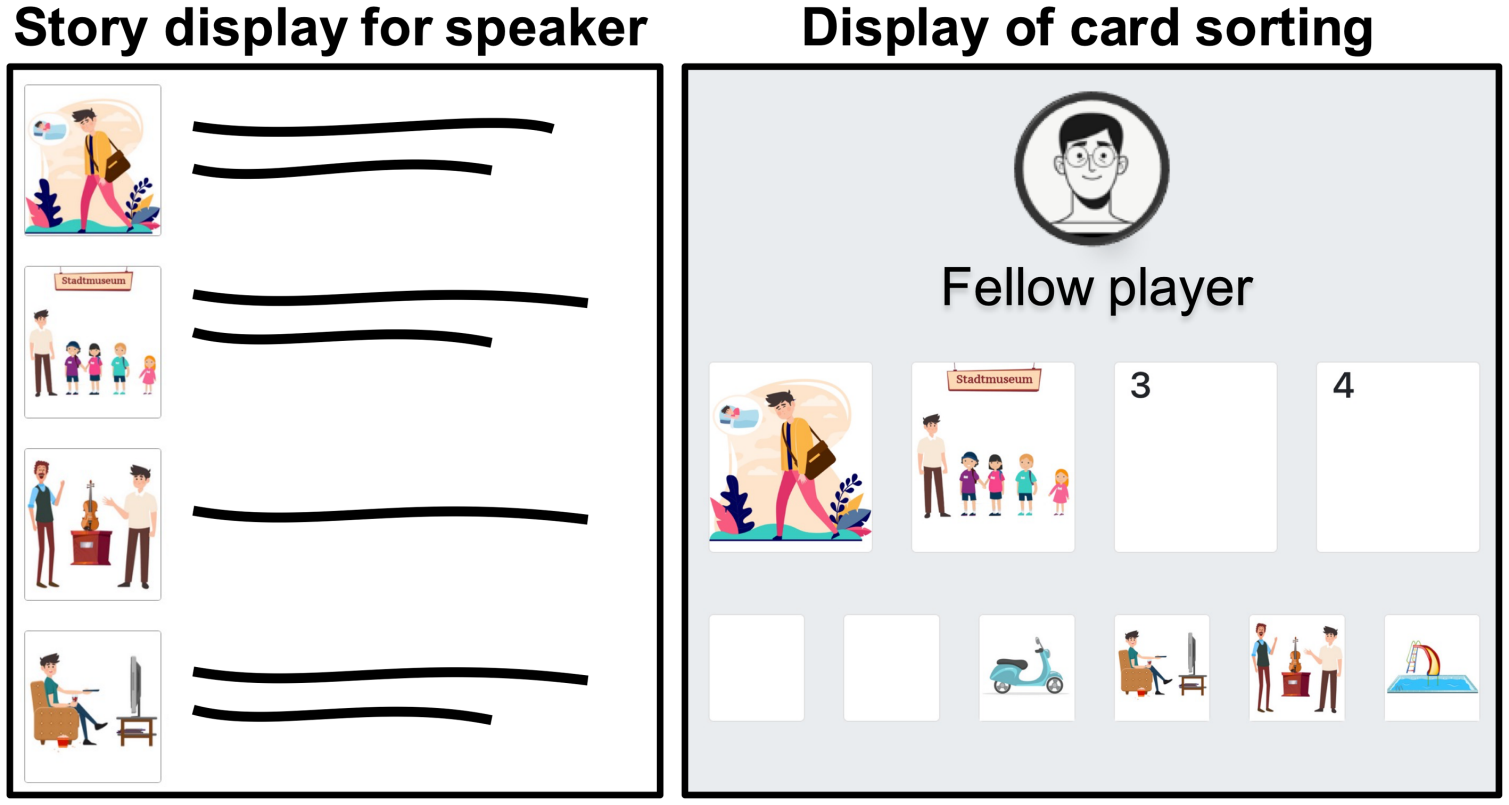
Schematic depiction of participant’s screen during the picture sorting task.

Recordings were collected remotely via a video call with the participant, the experimenter and a confederate in the summer of 2021, when COVID restrictions were still widely in place. To foster engagement and thus prevent monotonous speech, participants were asked to perform an interactive task together with the confederate, whom they thought to be another participant of the experiment. The task was implemented as an animated browser app based on a Flask server with SocketIO written in Python. Participants read a short story aloud in such a way that the confederate would be able to memorize the story and, after a short delay, sort corresponding picture cards (see Section 2.2) into the correct order. While reading the stories, participants saw both the story and the corresponding pictures in the correct order (see Figure 5, left panel). They were encouraged to first silently read and comprehend the story before reading it out loud. After reading, the participants were able to simultaneously watch the confederate sort the picture cards (see Figure 5, right panel) and provide feedback on the correctness of the order of the pictures after the task was finished. During the picture sorting task, an additional two pictures were presented that were clearly unrelated to the story, which served as distractors to make the task seemingly more difficult for the listener.

The sorting was pre-programmed and always resulted in the correct order. Before the actual task started, there were two example stories to practice the procedure. In cases of hesitations, repairs, exaggerated segmental articulation, or continuation rises produced by the participant in the target sentences, they were asked to repeat the reading and sorting task of the affected story at the end with the experimenter feigning technical difficulties.

During the interaction, each participant wore headphones and sat in a quiet room at home in front of their computer, which served as their recording device. The recording itself was controlled by the experimenter via the podcasting app Ennuicastr.^4^ Ennuicastr records participants directly onto their own devices on separate audio tracks, which prevents unstable internet connections from distorting the audio. Zoom’s built-in recording function was used as a back-up, which had to be used for 8 participants due to problems with the Ennuicastr recordings.

The use of Ennuicastr required minimal effort on the side of the participant. No prior installation of any software was needed since Ennuicastr runs directly in the browser. Participants joined the recording via a link that was created and sent to them by the experimenter. We recorded audio in lossless FLAC and downloaded the recordings in wav-format. However, the quality of recordings ultimately depended on the microphone quality on the side of the participant, which we were not able to control.

### 2.4 Data, annotation and measurements

Each participant read one version of each story, resulting in a dataset of 256 utterances including 512 target words. Recordings were force-aligned via WebMAUS (Schiel, 1999; Kisler et al., 2017) and segment boundaries were subsequently manually corrected in Praat (Boersma and Weenink, 2023). The suprasegmental annotation was conducted independently by two trained annotators (one of them the first author of the present study) following the DIMA guidelines (Kügler et al., 2022). DIMA is an annotation system rooted in the autosegmental-metrical (AM) framework of intonation analysis. It aims to be phonetically more transparent than other AM-based labeling systems (e.g., GToBI) thus facilitating annotation, but nevertheless reflects the phonological core of a contour. In cases of disagreement, a consensus was reached between the annotators and at least one other expert.

Specifically, two levels of phrase boundaries, strong (%) and weak (−), were annotated (agreement rate: 88%). Here, we consider the presence or absence of a phrase boundary after a target referent as a binary variable. Target utterances were frequently produced with a phrase boundary after Word1 (in 58% of cases) but rarely after Word2 (4%). On the tone tier, accentual and non-accentual tones were annotated, the latter as turning points occurring before and after pitch accents. For the accent type analysis, we translated the DIMA labels to GToBI (Grice et al., 2005) accent categories, for better comparability to previous studies (agreement rate: 89%).^5^ As a way to better compare the prominence of accent types across information status conditions that at the same time provides some perceptual validity, we assigned a prominence score to each accent type based on an independent prominence rating study by Baumann and Röhr (2015) with 68 native German listeners. In that study, participants rated the perceived degree of prominence of words on a visual analogue scale ranging from 1 to 100. The prominence scores reflect the average scores per accent type realized on the target word (summarized in Table 1).

**Table 1:** Summary of GToBI accent types and corresponding prominence scores following Baumann and Röhr (2015).

GToBI accent type	Accent type prominence score
L + H*	78.86
L* + H	71.53
H*	69.64
H +!H*	62.69
H + L*	57.14
!H*	53.62
L*	43.79

The scores represent mean ratings of perceptual prominence using a visual analogue scale ranging from 1 to 100.

Additionally, we measured several continuous phonetic parameters related to timing and the F0 contour. First, we measured the duration of the accented syllables of the target words in milliseconds. Target words that were audibly perturbed by internet connection issues (in cases in which we had to use the back-up Zoom signal) were excluded due to the duration measures being unreliable. In addition, phrase-final target words were not considered because of the potential effect of final lengthening. Furthermore, we included two measures from the ProPer toolbox (Albert et al., 2018; Albert, 2023), periodic energy mass and Delta F0. Both of these measures are based on periodic energy, which acoustically combines fundamental frequency and intensity by capturing the power of the periodic parts of the signal. This acoustic operationalization is motivated by perception, as periodic energy is correlated with pitch intelligibility (Albert, 2023, pp. 55–56).

**Figure 6:**
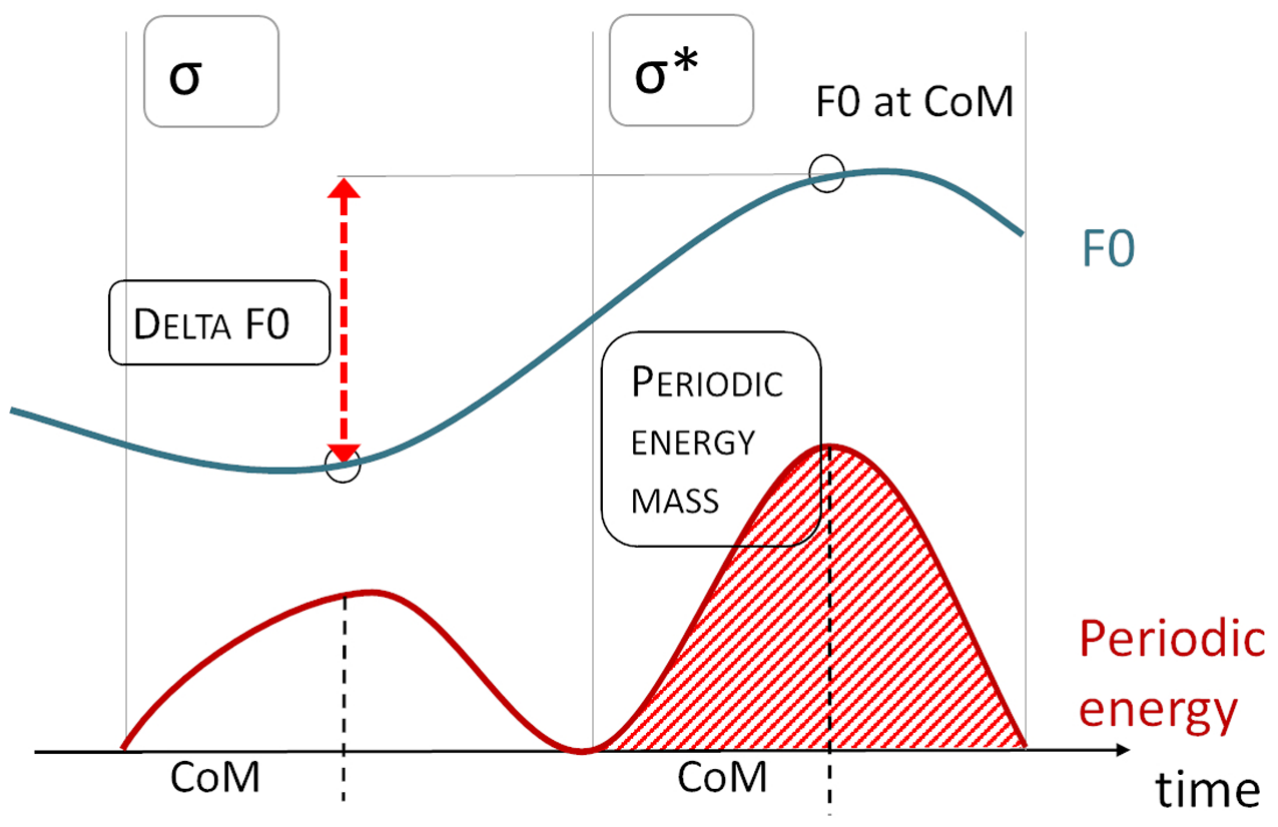
Schematic depiction of Delta F0 and Periodic Energy Mass measures from the ProPer toolbox (Albert, 2023).

Periodic energy mass quantifies the area under the periodic energy curve within an interval, typically the syllable (see Figure 6). It is the integral of duration and power. Since raw mass values are not deemed informative for various technical reasons (Albert, 2023, p. 150), relative mass is calculated by dividing the mass value for one syllable by the utterance’s average mass value per syllable. The resulting unitless values are centered around 1, with values below 1 indicating weak mass and values above 1 indicating strong mass. Distorted recordings and phrase-final target words again had to be excluded, since the mass measure is contingent on duration and thus could also reflect effects of phrase-final lengthening. Since relative mass is determined using the utterance’s average mass, we excluded both target words in an utterance that contained distorted audio.

Delta F0 captures the difference in semitones (st) between the F0 at the Center of Mass (CoM) of the accented syllable of a target word and the CoM of the syllable preceding it, thus reflecting F0 movement across syllables (see Figure 6). The CoM is the point in time that splits the area under the periodic energy curve of a syllable into two equal parts. Delta F0 is thus independent of landmark annotations and turning points, characterizing the F0 contour without the need for prior labeling. Table 2 summarizes all parameters considered here.

**Table 2:** Summary of the parameters measured.

Parameter	Operationalization
Accent type prominence score	(Pseudo-)continuous, reflects perceived accent type prominence (in percent)
Delta F0	Continuous, F0 difference between accented and preceding syllable (in semitones)
Phrase boundaries	Binary, presence of boundary after target word
Syllable duration	Continuous, duration of accented syllables in non-phrase-final referents (in milliseconds)
Periodic energy mass	Continuous, integrates duration and power of the accented syllable (unitless)

### 2.5 Statistics

We used Bayesian mixed effects linear regression models to investigate the paradigmatic and syntagmatic effects of information status on the prosodic realization of referents in two different positions. Note that the statistical analyses presented here are considered to have an exploratory character due to the complex nature of the research object and the relatively low token number.

We ran two models for each prosodic parameter, one to explore paradigmatic effects and one for the syntagmatic investigation. The prosodic parameter in question was always included as the response variable. The continuous phonetic parameters were *z*-scored. Phrase boundary was modeled as a binary variable.

For the investigation of the paradigmatic effects, each model included information status of the target word (levels: *new*, *accessible*) and position (levels: *Word1*, *Word2*) as well as their interaction as predictors. The predictors were coded with treatment contrasts and the reference levels were *accessible* and *Word1*. In addition, we included random intercepts for word and speaker and by-speaker random slopes for information status and position to account for individual differences in the usage of the different acoustic parameters.

Random slopes capture the direction and size of an effect for each individual, which makes them ideal for the analysis of speaker-specific behavior. We extracted the by-speaker random slopes for information status and ran a hierarchical cluster analysis on these, following Baumann and Winter (2018) who performed a similar analysis using frequentist regression models. The cluster analysis served to group together speakers who follow similar strategies in their encoding of information status. Rather than considering each of the 32 individuals separately, which quickly becomes convoluted, the amount of the strategies that need to be considered is thus reduced in an objective manner.

To explore potential syntagmatic effects, we ran separate models, where we included information status as a variable with four levels, categorizing the information status of both referents in the utterance, i.e., *[new, new]*, *[new, accessible]*, *[accessible, new]*, *[accessible, accessible]*. Again, position (levels: *Word1*, *Word2*) and the interaction of position and information status were included in the model. The reference levels for the treatment contrasts were *[accessible, accessible]* and *Word1*. We also included random intercepts for word and speaker and by-speaker random slopes for information status and position, similar to the paradigmatic models.

**Figure 7:**
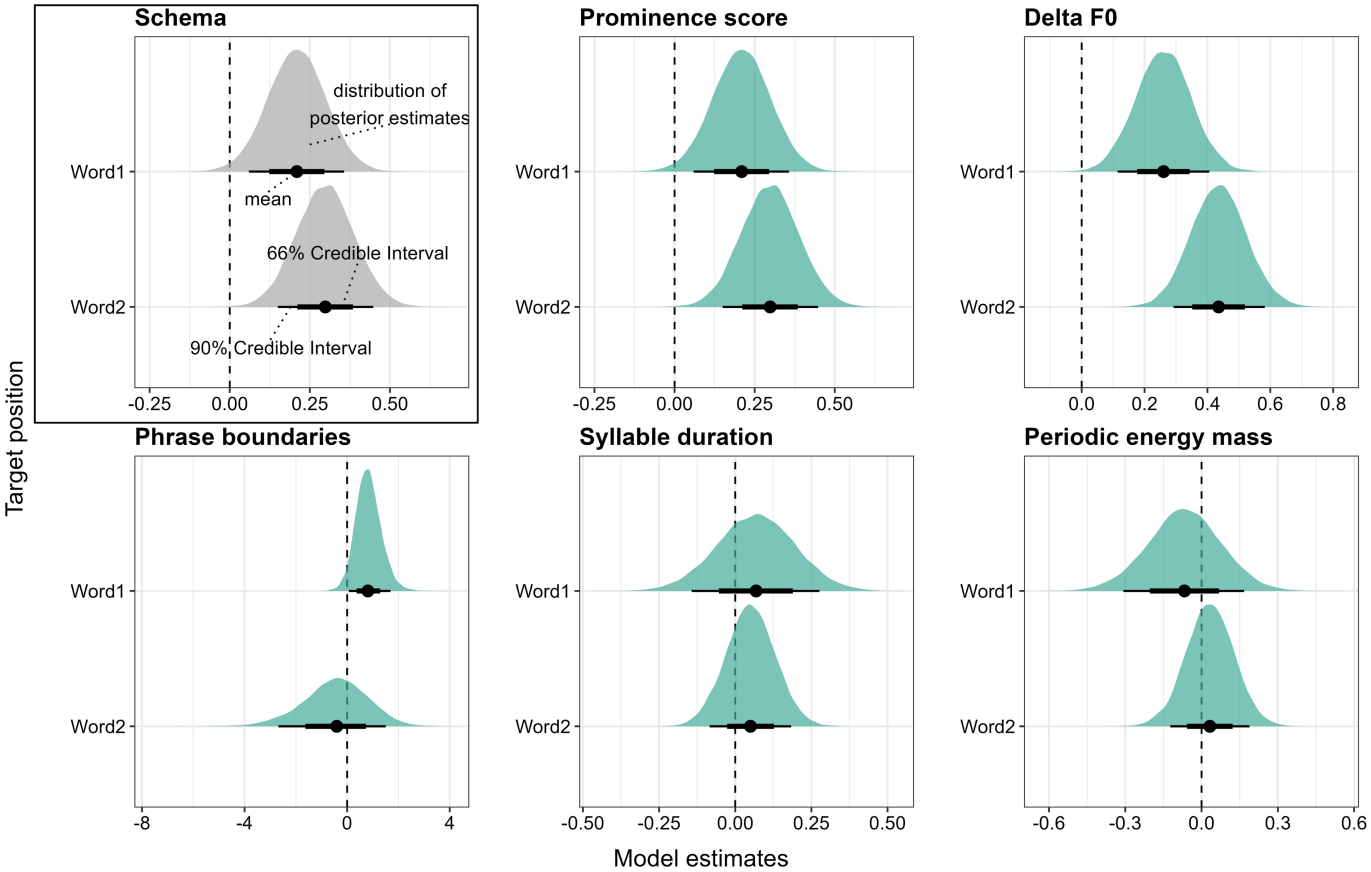
Posterior estimates for the five parameters as predicted by the models, means, 66% (thick horizontal lines) and 90% credible intervals (thin lines). Estimates show the change from *accessible* to *new* referents. Positive values indicate that the parameter is higher in *new* referents than in *accessible* ones.

The models were fitted in R (R Core Team, 2022) using the Stan modeling language (Carpenter et al., 2017) via the *brms* package (Bürkner, 2017). Four sampling chains ran for 11,000 iterations each with a warm-up period of 5,500 iterations, yielding a total of 22,000 posterior samples per model. We used a weakly informative, normally distributed prior with a mean of zero and a standard deviation of ten for the regression coefficients and default priors supplied by *brms* for the remaining parameters. Model fits were assessed visually by inspecting the posterior predictive checks and by ensuring that no model yielded *Rhat* values larger than one. For each model, we report the regression coefficient *β* and 90% credible intervals (*CI*s) under the posterior distributions as well as the posterior probability that *β* is larger than zero: *Pr(β > 0)*. If zero is not included in the *90% CI* and the posterior probability *Pr(β > 0)* is larger than 0.95 or smaller than 0.05, we consider there to be compelling evidence that *β > 0* or *β < 0*, respectively. Visually, the results of the models are depicted via half-eye plots generated using the *ggdist* package (Kay, 2023, see Schema in Figure 7). The colored density plots visualize the distribution of the posterior estimates, the thick and thin horizontal lines show 66 and 90% *CI*s, respectively, and the dot in the center of these lines represents the mean. The vertical dotted line marks the position where the estimate *β* equals zero, i.e., where there is no difference between the conditions that are being compared. When the thin horizontal black line does not cross the vertical zero line, the criterion that zero is not included in the *90% CI* is fulfilled.

## 3 Results

### 3.1 Paradigmatic effects

**Figure 8:**
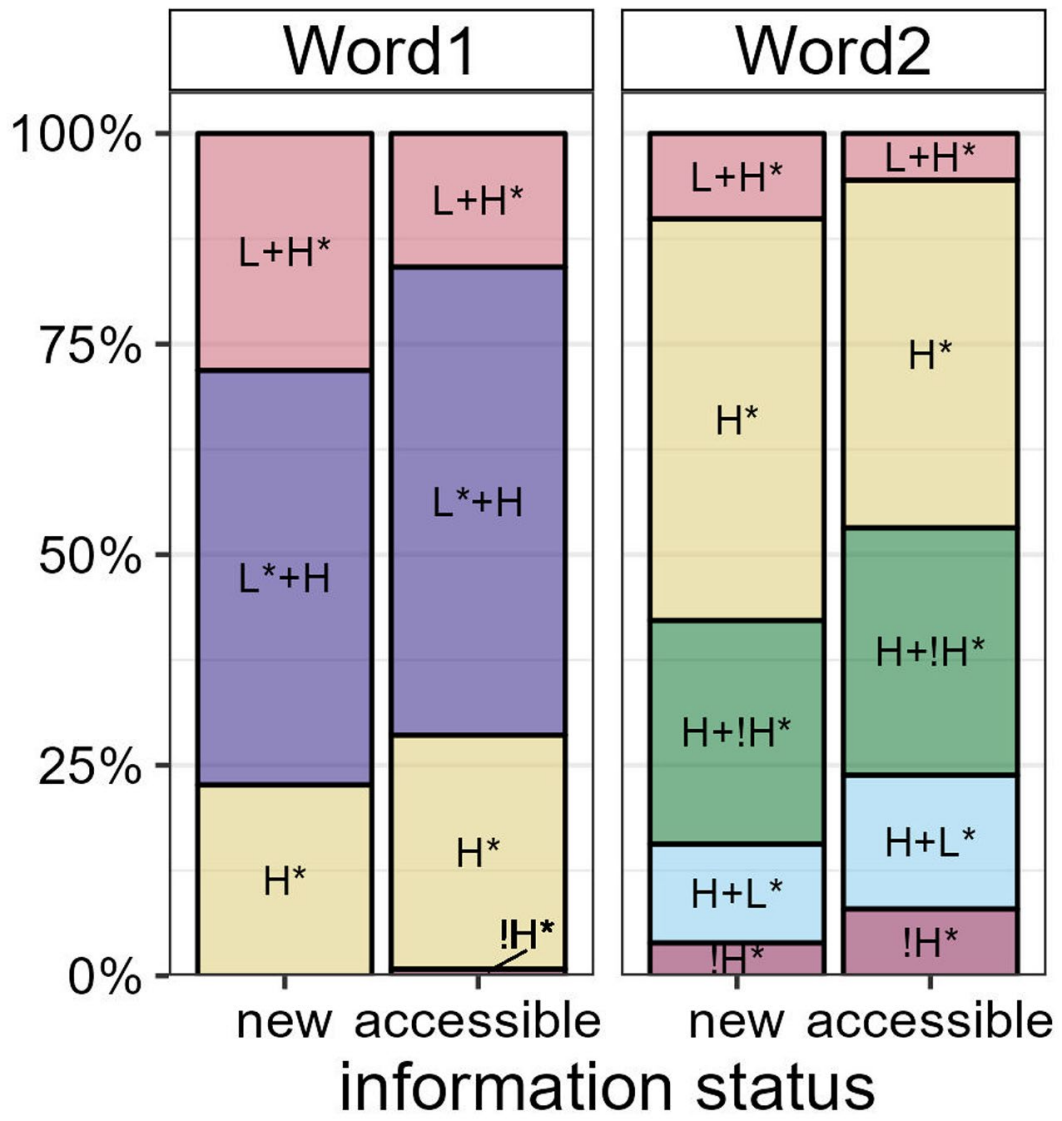
Proportions of GToBI accent types realized on Word1 and Word2 by information status.

We first present our findings on the paradigmatic effects of information status on the prosodic realization of referents. Target utterances were always produced in broad focus with prenuclear or nuclear pitch accents on the target words. The bar plots in Figure 8 show proportions of GToBI accent types as a function of information status in both Word1 and Word2.^6^ In Word1, rising accents such as L + H* and L* + H are produced predominantly, while falling accents such as H +!H* and H + L* occur only in Word2. In Word2, H* accents are the most frequent accent type. Accent types in both positions often combine to form a “hat pattern” (e.g., Féry, 1993).

In *new* words, there is a larger proportion of L + H* accents than in *accessible* words in both positions. In Word1, L* + H, H* and !H* accents (the latter occurring only once) are used more frequently in *accessible* than in *new* words. In Word2, H* is more frequent in *new* words, but H +!H*, H + L* and !H* accents are more frequent in *accessible* than in *new* words. Following the ranking in Baumann and Röhr (2015), *new* referents are thus generally marked by more prominent accent types than *accessible* referents.

Converting the accent types to a numerical scale, i.e., the accent type prominence scores collected by Baumann and Röhr (2015; see Section 2.4), yields mean values of 73.2 (sd = 3.65) for *new* and 71.8 (sd = 4.24) for *accessible* referents in the first position, and mean values of 66.6 (sd = 6.41) and 64.7 (sd = 6.68) for *new* and *accessible* referents, respectively, in the second position. The Bayesian mixed-effects model confirms that there is compelling evidence that *new* referents are produced with higher accent type prominence scores in both Word1 (*β* = 0.21, *CI* = [0.06; 0.36], *Pr(β > 0)* = 0.99) and Word2 (*β* = 0.30, *CI* = [0.15; 0.45], *Pr(β > 0)* = 1). Note that while mean prominence scores are higher in Word1 due to the presence of many high rising accents in this position, the effect of information status is more robust in Word2 (see Figure 7, top center).

Next, we consider Delta F0. This variable captures two out of three tonal dimensions that have been shown to be relevant for perceived prominence in German (e.g., Baumann and Röhr, 2015): the direction of pitch movement and degree of pitch excursion but not the height of the accentual tone. Here, in line with Baumann and Röhr (2015), we assume that high Delta F0 (i.e., steeply rising pitch) is perceived as most prominent, while low Delta F0 (i.e., steeply falling pitch) is least prominent. There is evidence that the relation between these tonal aspects and prominence is more complex, for example, a steeply falling H + L* accent is perceived as more prominent than a slightly rising !H* accent. However, !H* is rare in our dataset so that we can assume a simplified relation between Delta F0 and prominence in our data.

Referents in Word2 exhibit on average lower Delta F0 than those in Word1, which is due to the frequently falling contour in this position. Crucially for our research question, *new* referents are produced on average with higher Delta F0 values than *accessible* referents. In Word1, the mean Delta F0 is 1.17 st (sd = 1.99) in *new* target words and 0.60 st (sd = 1.68) in *accessible* ones. In Word2, *new* referents are produced with an average Delta F0 of −0.40 st (sd = 2.36) and *accessible* referents with −1.39 st (sd = 2.03). As evident from Figure 7 (top right), these differences are confirmed to be reliable by the Bayesian model for Word1 (*β* = 0.26, *CI* = [0.11; 0.41], *Pr(β > 0)* = 1) and Word2 (*β* = 0.44, *CI* = [0.29; 0.58], *Pr(β > 0)* = 1). Similar to the results for accent type prominence, the effect of information status is more robust in Word2.

Next, we consider phrase boundary placement as the presence or absence of a boundary after a target word. Phrase boundaries are commonly placed after the first target word (in 58% of cases overall) but rarely after Word2 (only 4%). In the first position, phrase boundaries are more frequent after *new* target words (62.5%) than after *accessible* ones (53.5%). The Bayesian mixed-effects model confirms a reliable tendency for *new* referents in Word1 to be followed by more phrase boundaries than *accessible* referents (*β* = 0.83, *CI* = [0.07; 1.69], *Pr(β > 0)* = 0.96, see Figure 7, bottom left). The scarcity of boundaries after Word2 does not allow for strong conclusions and we will thus disregard this position.

In terms of syllable duration, accented syllables are on average shorter in Word1 (mean = 223 ms, sd = 52) than in Word2 (mean = 254 ms, sd = 51). Crucially, information status does not appear to have a systematic effect on duration (see Figure 7, bottom center). In Word1, accented syllables in *new* referents are on average 222 ms (sd = 59 ms) long, and in *accessible* referents, they are 224 ms (sd = 45 ms) long. In Word2, accented syllables are 256 ms (sd = 49 ms) and 254 ms (sd = 53 ms) long in *new* and *accessible* conditions, respectively. The durational differences between *new* and *accessible* referents cannot be considered reliable, neither in Word1 (*β* = 0.07, *CI* = [−0.14; 0.28], *Pr(β > 0)* = 0.7) nor in Word2 (*β* = 0.05, *CI* = [−0.08; 0.18], *Pr(β > 0)* = 0.74).^6^

Finally, most mass values are larger than 1, indicating relatively strong mass, which is unsurprising considering that we measure mass in accented syllables occurring in a stretch of speech containing mostly unaccented syllables. Mass values are on average larger in Word2 (1.85) than in Word1 (1.54). There is no systematic difference between mass values in *accessible* and *new* target words (see Figure 7, bottom right). In Word1, *new* referents are produced with an average mass of 1.51 (sd = 0.48) and *accessible* referents with an average mass of 1.56 (sd = 0.48). In Word2, both *new* and *accessible* referents exhibit an average mass of 1.85 (with standard deviations of 0.57 and 0.59, respectively). These differences do not prove to be reliable, neither in Word1 (*β* = −0.07, *CI* = [−0.31; 0.17], *Pr(β > 0)* = 0.32) nor Word2 (*β* = 0.03, *CI* = [−0.12; 0.19], *Pr(β > 0)* = 0.64).

In summary, the overall results suggest that the paradigmatic contrast between *new* and *accessible* referents is reliably encoded via the F0 contour (i.e., GToBI accent type and Delta F0) and phrase boundary placement, but not via syllable duration or periodic energy mass.

### 3.2 Syntagmatic effects

Based on previous studies discussed in Section 1.2, we can assume that the production of prominence is also syntagmatically influenced by the context. That is, a referent may be more or less prominent depending on the prominence of a following or preceding referent. Here, we consider the information status of both referents in the utterance. Although this is an exploratory analysis and previous findings vary, we expect a balancing effect prior to our analysis, i.e., a *new* referent following or preceding an *accessible* referent should be more prominent than a *new* referent following or preceding another *new* referent (see Section 1.4). This expectation is formalized in the comparisons of the posterior estimates we conducted. Positive estimates will thus provide evidence in favor of the balancing effect, negative estimates support the radiating effect.

**Figure 9:**
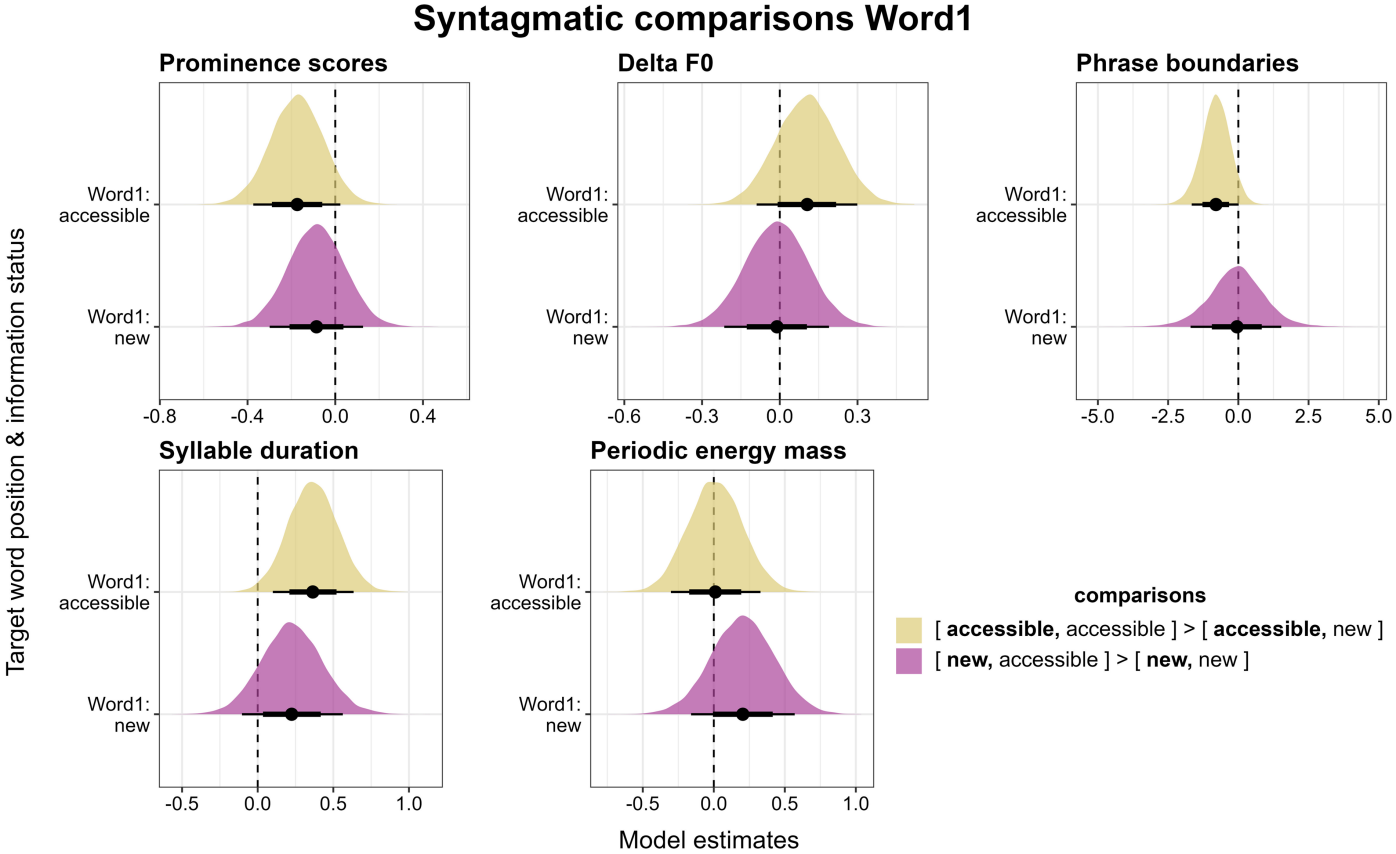
Posterior estimates for the five parameters as predicted by the models, means, 66% (thick horizontal lines) and 90% credible intervals (thin lines). Estimates show the changes for an *accessible* or a *new* referent in Word1 depending on whether it is followed by a *new* or an *accessible* referent. Positive values indicate that the parameter behaves according to our expectations (see “comparisons”).

We investigate syntagmatic effects by keeping the information status of Word1 or Word2 constant and comparing the realizations of these target words depending on whether they are followed/preceded by a *new* or *accessible* referent. First, we focus on Word1. We compare the first target word in the *[accessible, accessible]* condition to the first target word in the *[accessible, new]* condition to determine how *accessible* referents in this position are produced depending on whether they are followed by an *accessible* or a *new* referent. In Figure 9 (top left), we can observe that an *accessible* referent followed by a *new* referent is produced with more prominent accent types than if it is followed by an *accessible* word. This tendency is in line with the radiating effect, but it is not confirmed to be reliable by our model (*β* = −0.17, *CI* = [−0.37; 0.02], *Pr(β > 0)* = 0.07). Similarly, when comparing *new* referents in Word1 (i.e., the *[new, accessible]* and the *[new, new]* condition), there is evidence that those referents followed by other *new* referents are produced with more prominent accent types than those followed by *accessible* referents. This tendency is again not reliably predicted by the model (*β* = −0.09, *CI* = [−0.3; 0.13], *Pr(β > 0)* = 0.25).

For Delta F0, results with regard to syntagmatic effects are even less conclusive (see Figure 9, top center). *Accessible* referents followed by *accessible* referents are produced with higher Delta F0 values than *accessible* referents followed by *new* referents, which is in the expected direction of the balancing effect, however, this difference is not reliable according to the model (*β* = 0.1, *CI* = [−0.09; 0.3], *Pr(β > 0)* = 0.81). For *new* referents in Word1, there is barely a difference between those followed by other *new* and those followed by *accessible* referents (*β* = −0.01, *CI* = [−0.21; 0.19], *Pr(β > 0)* = 0.46).

Next, we consider phrase boundaries, focusing only on the presence or absence of a boundary after Word1, since boundaries are rare after Word2. Boundaries are least often placed in the *[accessible, accessible]* condition (48%), but equally often after *new* referents irrespective of whether they are followed by an *accessible* or another *new* referent (both 63%). The trend for more boundaries in the *[accessible, new]* (59%) than the *[accessible, accessible]* condition is expected under the radiating effect if boundary placement is considered to boost the prominence of the preceding word. It seems fairly robust, but is not quite reliable according to the model (*β* = −0.81, *CI* = [−1.66; 0.01], *Pr(β > 0)* = 0.05) (see Figure 9, top right). The difference between *new* referents followed by *accessible* or by *new* ones is clearly not reliable (*β* = −0.06, *CI* = [−1.7; 1.53], *Pr(β > 0)* = 0.48).

In terms of syllable duration, *accessible* referents in Word1 appear to be slightly longer when they are followed by another *accessible* referent than when followed by a *new* referent, which is in support of the balancing effect (Figure 9, bottom left). This difference is in fact reliable according to the model (*β* = 0.37, *CI* = [0.1; 0.63], *Pr(β > 0)* = 0.99). However, we need to caution at this point that this finding is based on very few data points (*n* = 32 for *[accessible, accessible]* and *n* = 25 for *[accessible, new]*) due to the exclusion of phrase-final referents. For *new* referents, we observe the same trend in that they are longer when followed by *accessible* referents than when followed by *new* referents. This difference, however, does not appear to be reliable according to the model (*β* = 0.23, *CI* = [−0.1; 0.56], *Pr(β > 0)* = 0.87).

Finally, periodic energy mass does not exhibit any systematic syntagmatic effects (Figure 9, bottom right). In Word1, *accessible* referents barely differ based on whether they are followed by a *new* or an *accessible* referent (*β* = 0.01, *CI* = [−0.3; 0.33], *Pr(β > 0)* = 0.52). *New* referents, on the other hand, seem to be slightly higher in mass when followed by an *accessible* referent than when followed by another *new* referent, which is in line with the balancing effect. However, this trend does not prove to be reliable (*β* = 0.2, *CI* = [−0.16; 0.57], *Pr(β > 0)* = 0.82).

**Figure 10:**
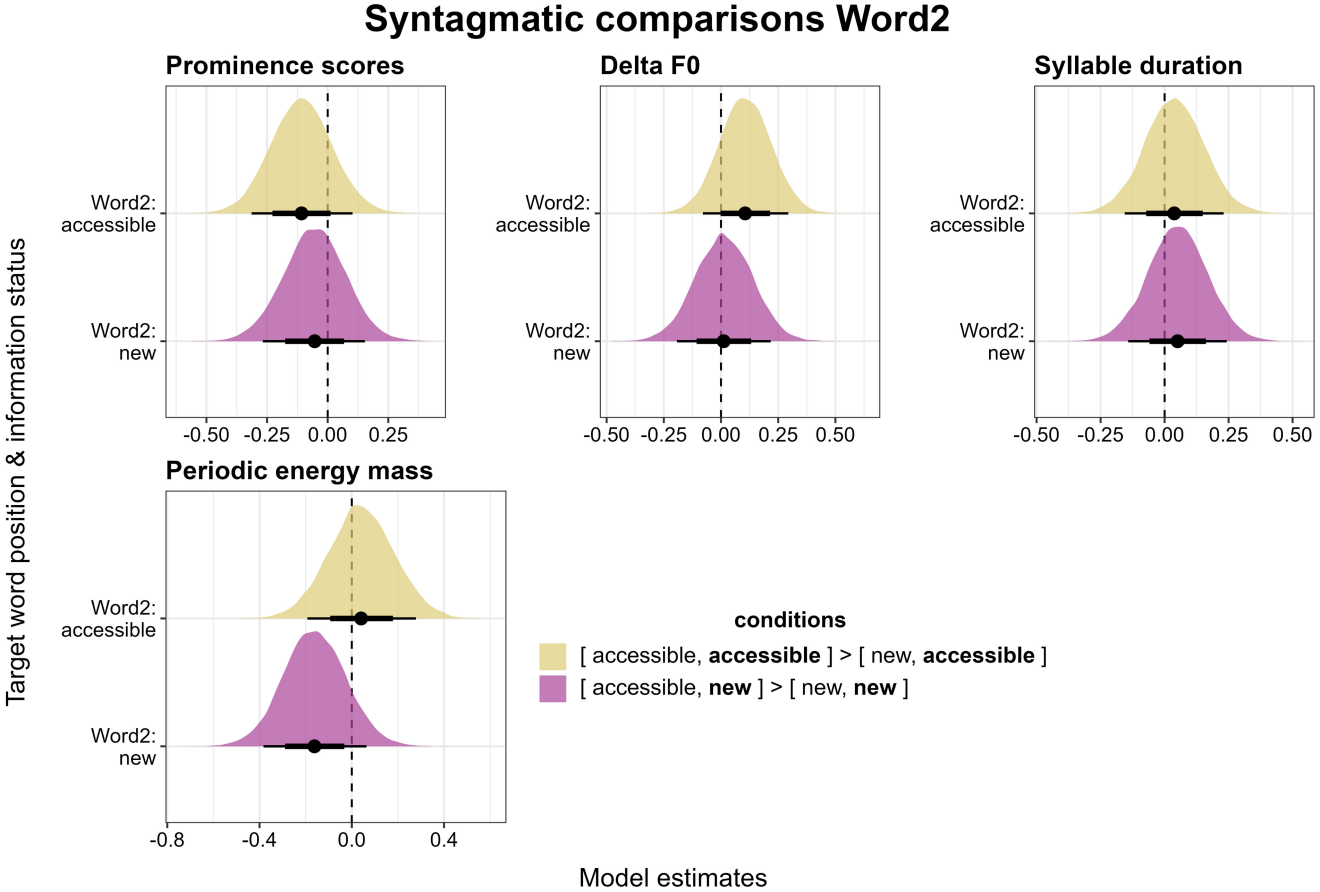
Posterior estimates for four parameters as predicted by the models, means, 66% (thick horizontal lines) and 90% credible intervals (thin lines). Estimates show the changes for an *accessible* or a *new* referent in Word2 depending on whether it is preceded by a *new* or an *accessible* referent. Positive values indicate that the parameter behaves according to our expectations (see “comparisons”).

Turning now to Word2 (Figure 10), we keep the information status of target words in this position constant and compare two words with the same information status preceded by either a *new* or an *accessible* word. The same trends as for Word1 can be observed regarding accent type prominence scores (Figure 10, top left). Referents preceded by *new* referents are produced with more prominent accent types than those preceded by *accessible* referents. This is true for both *accessible* referents (comparing *[accessible, accessible]* to *[new, accessible]*) and for *new* referents (comparing *[accessible, new]* to *[new, new]*). Again, these tendencies support the radiating effect, but neither difference proves to be reliable (for *accessible* Word2: *β* = −0.11, *CI* = [−0.31, 0.1], *Pr(β > 0)* = 0.2, for *new* Word2: *β* = −0.05, *CI* = [−0.27, 0.15], *Pr(β > 0)* = 0.33).

For Delta F0, again similarly to Word1, *accessible* referents preceded by *accessible* referents (*[accessible, accessible]*) are produced with slightly higher Delta F0 values than *accessible* referents preceded by *new* referents (*[new, accessible]*, Figure 10, top center). This trend goes in the expected direction of the balancing effect, however, it cannot be interpreted as reliable (*β* = 0.11, *CI* = [−0.08, 0.29], *Pr(β > 0)* = 0.83). Paralleling the findings for Word1, *new* referents in Word2 do not differ between the *[accessible, new]* and the *[new, new]* condition (*β* = 0.01, *CI* = [−0.19, 0.22], *Pr(β > 0)* = 0.54).

In Word2, there are no reliable differences in syllable duration between *accessible* referents preceded by *accessible* or *new* ones (*β* = 0.04, *CI* = [−0.16, 0.23], *Pr(β > 0)* = 0.63) nor between *new* referents preceded by *accessible* or *new* ones (*β* = 0.05, *CI* = [−0.14, 0.24], *Pr(β > 0)* = 0.67, Figure 10, top right). Finally, for periodic energy mass, there is again no difference between *accessible* referents preceded by *accessible* or *new* referents (*β* = 0.04, *CI* = [−0.19, 0.28], *Pr(β > 0)* = 0.62, Figure 10, bottom). However, *new* referents that are preceded by other *new* referents seem to have higher mass values than *new* referents preceded by *accessible* ones, which supports the radiating effect. This difference is not reliable (*β* = −0.16, *CI* = [−0.38, 0.06], *Pr(β > 0)* = 0.12).

In summary, we find barely any strong evidence to support our *a priori* expectations that *new* referents should be produced more prominently when occurring before or after an *accessible* referent than before or after another *new* referent, while *accessible* referents should be less prominent when followed or preceded by a *new* referent than by another *accessible* referent (which we call *balancing effect*). Only syllable duration in *accessible* words in position Word1 exhibits a reliable difference in the expected direction. We also find some support for the opposite tendency (termed *radiating effect*), which is weak, since most differences are not reliable according to our decision criteria, but still somewhat consistent: In all four information status combinations, *new* referents increase the prominence scores (derived from the pitch accent types used) of their neighboring referents, irrespective of whether they are *accessible* or *new*.

### 3.3 Individual strategies

**Figure 11:**
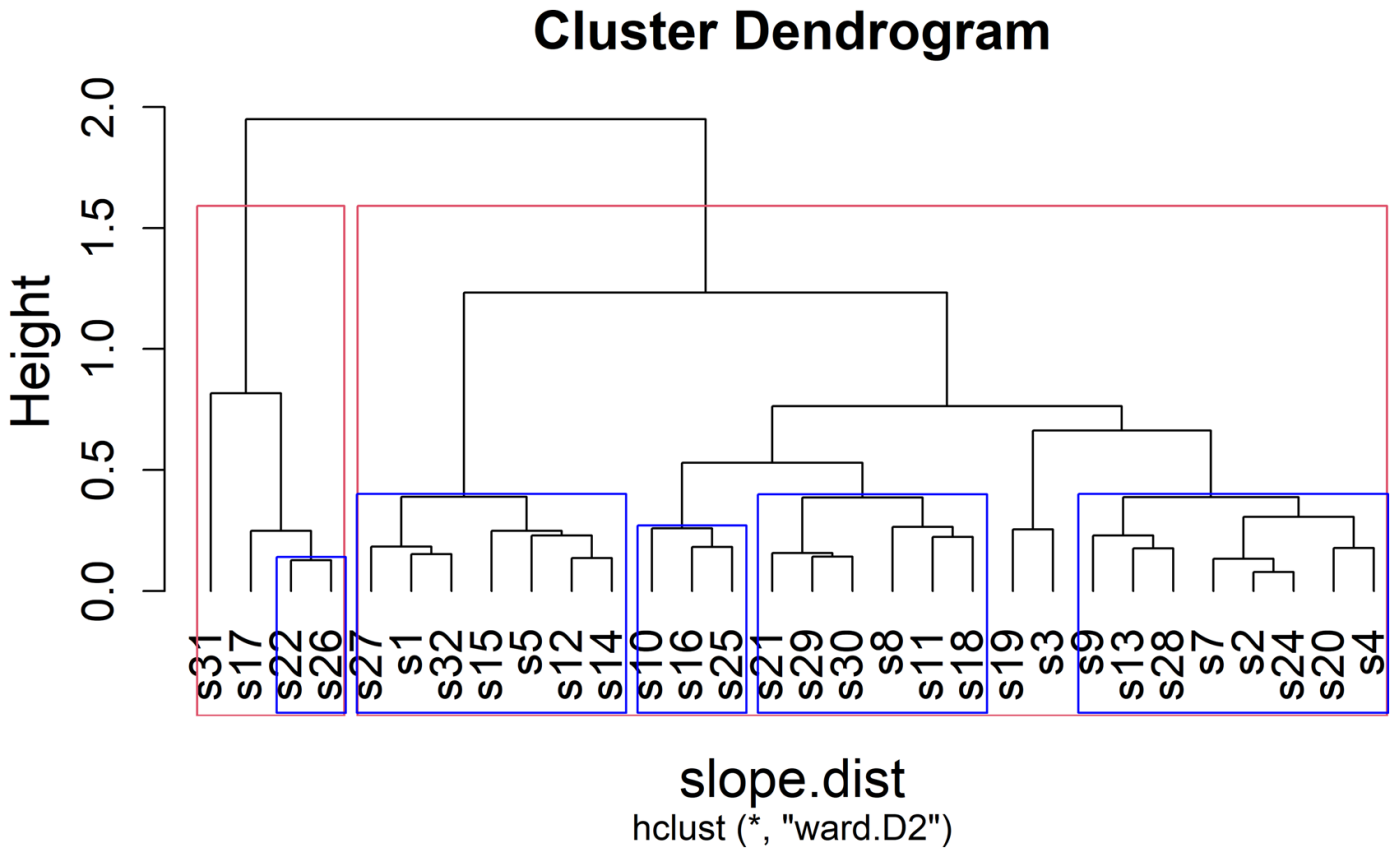
Results of cluster analysis. Red boxes: Clustering with highest average silhouette value. Blue boxes: Clusters that are supported by the data according to the resampling algorithm.

Previous research has often discovered inter-individual variability in the encoding and decoding of prosodic prominence (see Section 1.3). Participants of these earlier studies often cluster into groups of speakers or listeners employing the same strategies. In order to identify such strategies in information status marking across speakers, we extracted the by-speaker random slopes for the effect of information status from four of the models presented in Section 3.1 (i.e., only the models with a continuous dependent variable).^7^ We ran a cluster analysis on these estimates. Following Levshina (2015), who suggests that average silhouette width serves as an indicator of the optimal number of clusters, a two-cluster solution is initially deemed the best fit for our data (see Figure 11, red rectangles). To validate the stability of the clusters, we used multiscale bootstrap resampling in the package *pvclust* (Suzuki et al., 2019). Results indicate that five sub-clusters (see Figure 11, blue rectangles) of our initial two clusters are supported by the data.^8^ In the following paragraphs, we will thus report on the characteristics of the five sub-clusters.

**Figure 12:**
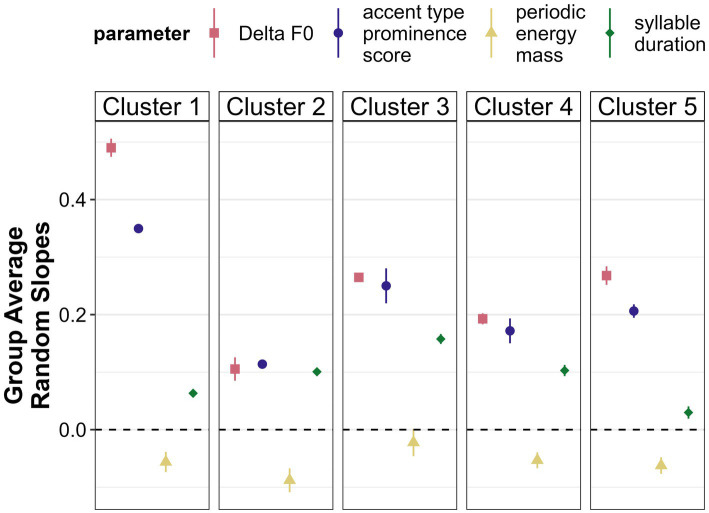
Mean random slopes and standard errors per cluster. The larger the value, the more strongly the cluster marks information status via this parameter in the expected direction.

Figure 12 shows the averaged random slopes for each of the five clusters in comparison to the overall results. A positive value here indicates that this parameter is higher in *new* referents than in *accessible* ones and is thus employed to mark the information status contrast in the expected direction, a value around zero indicates that there is no difference and negative values indicate higher values in *accessible* referents as opposed to *new* ones.

Rather uniformly, the F0-related parameters emerge as the most important correlates of information status among all clusters. Periodic energy, however, is not used consistently in the expected direction by any cluster. With regard to duration, there is some variability across clusters. While in Cluster 5 (eight speakers), there is barely a difference in terms of syllable duration between *new* and *accessible* referents, Cluster 3 (three speakers) does use duration as a cue, at least to a larger extent than all other clusters. In Cluster 2 (seven speakers), syllable duration is as strong a cue as Delta F0 and accent type prominence score.

Clusters also differ in regard to the strength of encoding. While Cluster 1 (two speakers) subsumes particularly strong encoders, the remaining clusters distinguish between *new* and *accessible* referents much less clearly. Especially Cluster 2 seems to only weakly mark a difference in information status. Cluster 3 and Cluster 4 (six speakers) are very similar in their ranking of parameters, only differing in strength of encoding with Cluster 4 containing the weaker encoders.

It appears that the initial two-way split between speakers s22 and s26 (Cluster 1) plus two other speakers and the remaining speakers pertains primarily to the *strength* of encoding of the information status contrast. Looking at smaller sub-clusters allows us to discover more fine-grained differences between groups of speakers and another dimension of variability, namely the *type* of parameters used to encode information status.

Individual speakers thus seem to differ among two dimensions, namely the strength of encoding of the *new*-*accessible* contrast and the choice of cues. However, most speakers make use of Delta F0 or accent type to encode information status.

## 4 Discussion

The innovative recording paradigm employed here enables us to investigate the prosodic encoding of information status in controlled yet less sterile material than usually elicited in experimental research. In addition, the paradigm provides an example of remote data collection in which participants are involved in an interactive task. Our results first and foremost confirm previous findings that information status is encoded via prosodic prominence in German, in that *new* referents are produced more prominently than *accessible* referents – which at the same time confirms our expectations regarding RQ1 on the paradigmatic perspective of the relation between information status and its prosodic marking. Our results are in line with the Smooth Signal Redundancy Hypothesis (Aylett and Turk, 2004; see Section 1.1), both at a phonological and a phonetic level. That is, *accessible* referents, which have become predictable through the discourse context, are produced with an attenuated acoustic signal, reflected not only by less prominent accent types and fewer insertions of prosodic boundaries after the target word but also by generally smaller F0 changes. *New* referents, on the other hand, are contextually unpredictable and thus produced here with relatively more prominent - and on average more steeply rising - accents, and are more often followed by a prosodic boundary, supposedly further enhancing the prominence of the preceding lexical item. Interestingly, other (continuous) measures such as duration or periodic energy, which have been shown to contribute to the encoding of prominence in various languages, were not modulated by the information status contrast. These findings further underline the precedence of F0 as a cue to phrase-level prominence (e.g., Baumann and Winter, 2018; Bishop et al., 2020; Roessig et al., 2022).

Turning to RQ2, we looked at the potential influence of the referents’ information status on the prosodic prominence relation between them. As outlined in Section 1.2, syntagmatic effects are less well understood and different studies report conflicting findings. Our own syntagmatic findings are also less conclusive than our paradigmatic ones. A compelling previous finding that we expected to confirm prior to our analysis is that of a *balancing* or trade-off effect as, for example, observed by Rump and Collier (1996) or Roessig (2024). Here, prominence is distributed across referents in an utterance from a fixed budget: As the prominence of one referent increases, the prominence of another referent in the same utterance decreases. However, we observed this effect only in syllable duration and only reliably in one out of four comparisons. As for choice of pitch accent type and phrase boundary insertion, we find a different tendency, which was weaker yet more consistent: The presence of a prominent (i.e., *new*) referent in an utterance raises the prominence of another (preceding or following) referent in the same utterance, irrespective of its information status. This tendency is reminiscent of the Gussenhoven-Rietveld Effect, which describes that a more prominent pitch accent raises the perceived prominence of a following pitch accent (Gussenhoven and Rietveld, 1988). For the purpose of the present study, we refer to our comparable observation on the production side as a *radiating effect*, a term which is agnostic as to the direction of the process.

**Figure 13:**
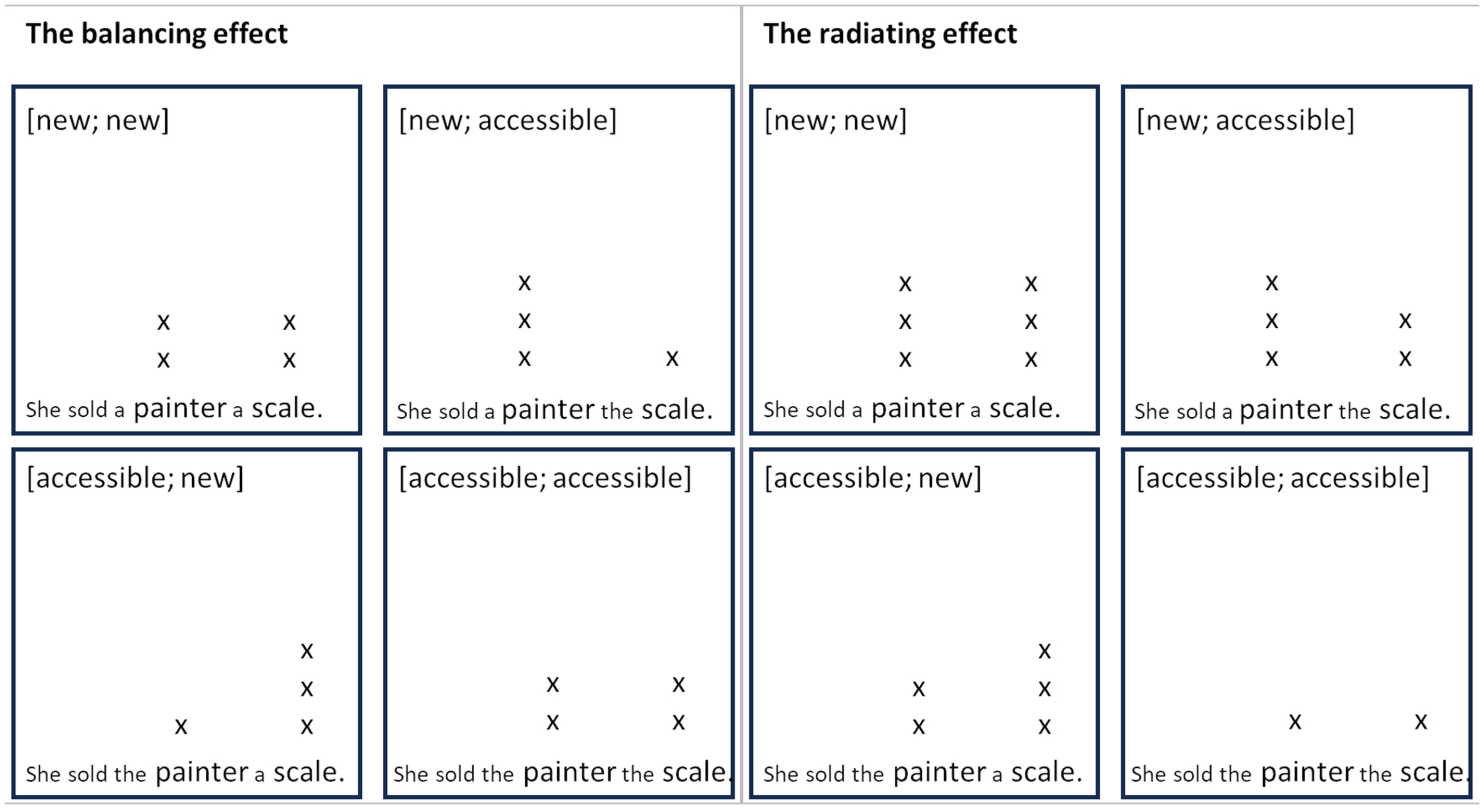
Metrical grids of balancing versus radiating effects in all four conditions.

The contradicting findings of balancing versus radiating effects are systematized in the metrical grids in Figure 13. Metrical grids traditionally were not intended to capture subtle prosodic differences between pragmatic categories such as *new* and *accessible* referents (e.g., Liberman, 1975; Hayes, 1995). However, we make use of the flexibility they allow in the number of layers of beats to represent prominence relations between entities. Under the balancing effect, the number of beats assigned to all referents is equal in every single utterance (see Figure 13, left). The redistribution of prominence is indicated by the movement of a beat from a less prominent to a more prominent referent in the *[accessible, new]* or *[new, accessible]* condition as compared to the *[new, new]* or *[accessible, accessible]* condition, where the prominence relation between the two referents is balanced. Operating under the assumption of a fixed number of beats also implies that referents in *[new, new]* utterances are equally prominent as referents in *[accessible, accessible]* utterances. However, this is not what we observe in our data, as both referents in the *[new, new]* condition are realized more prominently than the referents in the *[accessible, accessible]* condition. Similarly, Rump and Collier (1996) find that both referents in double contrastive focus are more prominent than the referents in broad focus (see Figure 3 in Section 1.2). These observations are accounted for in the radiating effect by the addition of beats to more prominent referents (Figure 13, right). The presence of a *new*, i.e., a more prominent referent in the utterance raises the prominence of both referents in the utterance, so that a beat is added to both referents in the *[new, accessible]* and the *[accessible, new]* conditions in comparison to the *[accessible, accessible]* condition. In order to keep the prominence relation between *new* and *accessible* referents tipped in favor of the *new* referent, another beat is added to the *new* referent in these conditions. In the *[new, new]* condition, the radiating effect raises the prominence and thus the number of beats in both referents simultaneously and to the same degree. That is, the radiating effect gives more weight to the paradigmatic influence of the respective semantic-pragmatic contrasts (in our case *new* vs. *accessible* information). In this respect, the radiating effect is not purely syntagmatic in nature, at least not to the same extent as the balancing effect is. In any case, both effects can be expected to occur in combination, and this is what we seem to find in our dataset as well.

The metrical grids represent an abstraction of the two effects observed in our data and in different previous studies. While the distribution of different layers of beats allows for some granularity in the representation of prominence as opposed to a binary branching tree (see Figure 2 in Section 1.1), one could ask whether the grids are fine-grained enough to capture the subtle changes in the realizations of referents we can attribute to either balancing or radiating effects in our data. The alternative is a continuous or gradient representation. This choice relates to a broader discussion in prosodic research on the categoriality and gradience of the suprasegmental signal (see, e.g., Grice et al., 2017; Roessig, 2021; Ladd, 2022).

In any case, the syntagmatic effects we observe are subtle. In fact, many contrasts do not reach the reliability threshold according to our statistical models. A potential explanation lies in the fact that most previous findings regarding syntagmatic effects of prominence, supporting either the balancing or the radiating effect, are based on perception experiments (Gussenhoven and Rietveld, 1988; Ladd et al., 1994; Rump and Collier, 1996). These syntagmatic effects may to a large extent reflect a perceptual illusion and not so much an actual encoding in the signal. However, Roessig (2024) in a recent production study has found a balancing effect for both F0 and duration comparing two subsequent referents in two different focus structures: When the first referent is in the background and is followed by a referent in contrastive focus, the difference between the two referents is greater compared to a condition where the first referent in the background is followed by narrow focus (i.e., background becoming less and focus becoming more prominent). Our exploratory data contribute new insights to this question by exploring the syntagmatic effects of another pragmatic contrast, i.e., information status, and finding tentative support for both effects. A question that arises is, thus, under which circumstances do balancing or radiating effects emerge? Beside linguistic manipulations of meaning, work by Ladd et al. (1994) suggests that paralinguistic meaning may play a role. Much work remains for future studies considering larger sample sizes and more varied contexts as well as pragmatic contrasts.

Finally, regarding RQ3, we investigated individual differences in the prosodic encoding of information status. One reason for the large degree of variability observed in prosodic data is redundancy in the signal (e.g., Winter, 2014). Acoustically, prosodic prominence is encoded via F0 movements, duration differences and intensity fluctuations and it is typically assumed that all three dimensions play a role in the encoding of certain (pragmatic) contrasts such as focus or information status in West Germanic languages. Redundant encoding in speech guarantees communicative success in noisy situations (Winter, 2014). In the present study, we explored how this redundancy allows for inter-speaker variability. In our data, the information status contrast is marked, on the group level, via parameters related to the F0 contour, both categorically in the choice of pitch accent type and continuously in the extent of F0 change (i.e., Delta F0) as well as via phrase boundary placement but not via duration or periodic energy. On the individual level, we find that most speakers clearly prefer F0-related cues, yet some speakers also rely on duration (albeit to a lesser extent), thus redundantly encoding prominence via two acoustic dimensions. However, no speaker encodes information status maximally redundantly, since periodic energy is never modulated in the expected direction. In fact, some speakers seem to solely rely on F0 modulations, i.e., they cannot be said to produce acoustically redundant signals.

The overarching preference for F0-related parameters we observe in our speakers corresponds to findings in perception studies. Listeners also seem to pay most attention to F0-related cues in their interpretation of prosodic prominence. For instance, in Baumann and Winter’s (2018) study, 18 listeners are identified to belong to the pitch-driven group while only 9 listeners attend more to lexical and semantic-syntactic cues as well as duration. Since our data was collected in a reading task where the exact wording of the utterances including the type of determiner (definite vs. indefinite) was prescribed as a morpho-syntactic cue to information status, we cannot conclude that a group of speakers relied more heavily on this cue.

The most salient difference between groups of speakers we observe is related to the strength of encoding. A couple of speakers produce a very strong contrast, while some speakers mark information status only relatively weakly. In perception studies, sensitivity to prosodic focus type distinctions has been linked to the concept of “pragmatic skill” or empathy (Bishop, 2016, 2017; Orrico et al., 2023). Analogously, we could expect speakers with higher pragmatic skill to encode information status contrasts more clearly than speakers with lower pragmatic skill. This expectation presumes a listener-oriented theory of speech production (*cf.* Turnbull, 2019). Alternatively, the difference in strength of encoding could arise from variability in the interpretation of *accessibility* on the side of the speakers. If the speakers did not interpret the *accessible* referents as predictable through the context, they may not have intended to produce this contrast prosodically. However, the use of indefinite articles for *new* referents and definite articles for *accessible* referents should have prompted the desired interpretation.

The robustness of the prosodic encoding of information status arguably has direct implications for perception: The more redundantly a speaker marks a given contrast in production, the more easily the listener can decode the message (Cangemi et al., 2015). However, it is unclear how the listener deals with contradictory prominence cues, e.g., when a speaker marks *new* referents as more prominent than *accessible* ones via the F0 contour, but at the same time *accessible* referents are higher in periodic energy than *new* ones (or vice versa). Another interesting question is whether modulating one parameter strongly or modulating several parameters moderately makes communication more robust. A corresponding perception study is needed to reveal the effectiveness of different prominence marking strategies.

## 5 Conclusion

The present study allows for new insights regarding the redundant and relational nature of prosodic prominence: (i) Paradigmatic effects prevail over syntagmatic ones in the encoding of information status. *New* referents are marked as more prominent than *accessible* referents, mainly by employing F0-related cues. (ii) In the context of the utterance, we observe both a balancing effect of prominence in terms of syllable duration and a radiating effect by which prominent entities appear to raise the prominence level of adjacent entities in terms of pitch accent type and phrase boundary placement. However, all observed effects are relatively small. (iii) There is substantial inter-speaker variability, especially regarding the strength of the prosodic encoding of information status. The apparent redundancy of prominence marking often observed at the group level arises partly through different individuals prioritizing different prosodic cues.

## Data availability statement

The datasets presented in this study can be found in online repositories. The names of the repository/repositories and accession number(s) can be found below: https://osf.io/frvm7/.

## Ethics statement

The studies involving humans were approved by Ethics Committee of the German Linguistic Society (Deutsche Gesellschaft für Sprachwissenschaft, DGfS). The studies were conducted in accordance with the local legislation and institutional requirements. The participants provided their written informed consent to participate in this study.

## Author contributions

JL: Conceptualization, Data curation, Formal analysis, Writing – original draft, Writing – review & editing. SR: Conceptualization, Data curation, Formal analysis, Writing – review & editing. SB: Conceptualization, Data curation, Writing – review & editing.

## Footnotes

^1^ https://corpora.uni-leipzig.de?corpusId=deu_news_2021

^2^According to an annotation following the RefLex scheme (Baumann and Riester, 2012; Riester and Baumann, 2017), our *accessible* referents would be classified as *r-unused* (r = referential level) and *l-accessible* (l = lexical level). *New* target words would be annotated as *r-new* and *l-new*.

^3^Picture attributions for the pictures in Figure 4: Living room, scale, hand - designed by Freepik; music notes - designed by brgfx/Freepik, painter, woman - designed by pikisuperstar/Freepik; radio, store, construction site - designed by macrovector/Freepik.

^4^ https://ecastr.com/

^5^Note that these annotations are based on the DIMA tone labels, for which a consensus was already reached prior to the GToBI annotations.

^6^Note that for better readability, we have collapsed L + ^H* with L + H* and ^H* with H* accents. Furthermore, we excluded L* accents, which occurred only twice in the entire dataset.

^7^Note that due to the exclusion of phrase-final referents, these findings are based on only 105 data points for Word1 and 238 for Word2.

^8^This analysis was conducted on a subset of 30 speakers, since for two speakers no slopes for syllable duration and periodic energy mass could be estimated due to the exclusion of data points.
