# Supplementary material for: Paradigmatic and syntagmatic effects of information status on prosodic prominence – evidence from an interactive web-based production experiment in German
Source: Front Psychol. 2024 Apr 9;15:1296933. doi: 10.3389/fpsyg.2024.1296933 (PMC11035868; doi:10.3389/fpsyg.2024.1296933)
Supplement: Supplementary file 1 [file Data_Sheet_1.PDF]

Supplementary Material

Paradigmatic and syntagmatic effects of information status on prosodic prominence – Evidence from an interactive web-based production experiment in German

Janne Lorenzen[1*], Simon Roessig[2], Stefan Baumann[1]

[1]IfL-Phonetik, University of Cologne, Cologne, Germany

[2]Department of Language and Linguistic Science, University of York, York, United Kingdom

*** Correspondence:**
Janne Lorenzen
janne.lorenzen@uni-koeln.de

1 Reading material

Story 1 (Lehrer *teacher* – Geige *violin*)

NEW-NEW:

1. Peter ist richtig müde von der Arbeit.
2. Er hat den ganzen Tag im Fundbüro für Besucher Gegenstände herausgesucht.
3. Unter anderem hat er einem Lehrer eine Geige gezeigt.
4. Jetzt ruht er sich vor dem Fernseher aus.

NEW-ACCESSIBLE:

1. Peter ist richtig müde von der Arbeit.
2. Er hat heute dutzende Besucher durch die kleine Instrumentensammlung im Stadtmuseum geführt.
3. Unter anderem hat er einem Lehrer die Geige gezeigt.
4. Jetzt ruht er sich vor dem Fernseher aus.

ACCESSIBLE-NEW:

1. Peter ist richtig müde von der Arbeit.
2. Er hat heute eine Schulklasse durch das historische Stadtmuseum geführt.
3. Unter anderem hat er dem Lehrer eine Geige gezeigt.
4. Jetzt ruht er sich vor dem Fernseher aus.

ACCESSIBLE-ACCESSIBLE:

1. Peter ist richtig müde von der Arbeit.
2. Er hat heute eine Schulklasse durch die kleine Instrumentensammlung im Stadtmuseum geführt.
3. Unter anderem hat er dem Lehrer die Geige gezeigt.
4. Jetzt ruht er sich vor dem Fernseher aus.

Story 2 (Nonne *nun* – Mühle *mill*)

NEW-NEW:

1. Tom ist richtig müde von der Arbeit.
2. Er hat den ganzen Tag Touristen durch das Stadtmuseum geführt.
3. Unter anderem hat er einer Nonne eine Mühle gezeigt.
4. Jetzt setzt er sich zum Entspannen in seinen Lieblingssessel.

NEW-ACCESSIBLE:

1. Tom ist richtig müde von der Arbeit.
2. Er hat heute dutzende Besucher durch das Brotmuseum geführt.
3. Unter anderem hat er einer Nonne die Mühle gezeigt.
4. Jetzt setzt er sich zum Entspannen in seinen Lieblingssessel.ACCESSIBLE-NEW:

1. Tom ist richtig müde von der Arbeit.
2. Er hat heute eine Gruppe aus der Klosterschule durch das Stadtmuseum geführt.
3. Unter anderem hat er der Nonne eine Mühle gezeigt.
4. Jetzt setzt er sich zum Entspannen in seinen Lieblingssessel.

ACCESSIBLE-ACCESSIBLE:

1. Tom ist richtig müde von der Arbeit.
2. Er hat heute eine Gruppe aus der Klosterschule durch das Brotmuseum geführt.
3. Unter anderem hat er der Nonne die Mühle gezeigt.
4. Jetzt setzt er sich zum Entspannen in seinen Lieblingssessel.

Story 3 (Anwalt *lawyer* – Säge *saw*)

NEW-NEW:

1. Jana ist richtig müde von der Arbeit.
2. Sie hat den ganzen Tag im Fundbüro für Kunden Gegenstände herausgesucht.
3. Unter anderem hat sie einem Anwalt eine Säge gezeigt.
4. Jetzt setzt sie sich mit einem Buch auf ihr Sofa.

NEW-ACCESSIBLE:

1. Jana ist richtig müde von der Arbeit.
2. Sie hat heute dutzende Besucher durch die kleine historische Werkzeugsammlung im Museum geführt.
3. Unter anderem hat sie einem Anwalt die Säge gezeigt.
4. Jetzt setzt sie sich mit einem Buch auf ihr Sofa.

ACCESSIBLE-NEW:

1. Jana ist richtig müde von der Arbeit.
2. Sie war heute bei einem Gerichtsprozess für die Beweisstücke zuständig.
3. Unter anderem hat sie dem Anwalt eine Säge gezeigt.
4. Jetzt setzt sie sich mit einem Buch auf ihr Sofa.

ACCESSIBLE-ACCESSIBLE:

1. Jana ist richtig müde von der Arbeit.
2. Sie war heute bei einem Gerichtsprozess für die Beweisstücke zum Mord im Baumarkt zuständig.
3. Unter anderem hat sie dem Anwalt die Säge gezeigt.
4. Jetzt setzt sie sich mit einem Buch auf ihr Sofa.

Story 4 (Bauer *farmer* – Birne *pear*)

NEW-NEW:

1. Nach zwei regnerischen Wochen schien endlich wieder die Sonne.
2. Maria hat sich ihre Schuhe angezogen und ist spazieren gegangen.
3. Unterwegs hat sie einem Bauern eine Birne geschenkt.
4. Jetzt ist sie wieder zu Hause und kocht sich einen Tee.

NEW-ACCESSIBLE:

1. Nach zwei regnerischen Wochen schien endlich wieder die Sonne.
2. Nachdem Maria ein paar Früchte eingesteckt hatte, ist sie spazieren gegangen.
3. Unterwegs hat sie einem Bauern die Birne geschenkt.
4. Jetzt ist sie wieder zu Hause und kocht sich einen Tee.

ACCESSIBLE-NEW:

1. Nach zwei regnerischen Wochen schien endlich wieder die Sonne.
2. Maria hat sich ihre Schuhe angezogen und ist zwischen den Feldern des benachbarten Gutshofs spazieren gegangen.
3. Unterwegs hat sie dem Bauern eine Birne geschenkt.
4. Jetzt ist sie wieder zu Hause und kocht sich einen Tee.

4: ACCESSIBLE-ACCESSIBLE

1. Nach zwei regnerischen Wochen schien endlich wieder die Sonne.
2. Nachdem Maria ein paar Früchte eingesteckt hatte, ist sie zwischen den Feldern des benachbarten Gutshofs spazieren gegangen.
3. Unterwegs hat sie dem Bauern die Birne geschenkt.
4. Jetzt ist sie wieder zu Hause und kocht sich einen Tee.

Story 5 (Maler *painter* – Waage *scale*)

NEW-NEW:

1. Anna hatte einen erfolgreichen Tag.
2. Sie hat die Kunden auf dem Flohmarkt für viele ihrer Waren begeistern können.
3. Unter anderem hat sie einem Maler eine Waage verkauft.
4. Jetzt ist sie zu Hause und entspannt sich bei schöner Musik.

NEW-ACCESSIBLE:

1. Anna hatte einen erfolgreichen Tag im Geschäft.
2. Sie hat die Kunden für die Restbestände bei den Küchengeräten begeistern können.
3. Unter anderem hat sie einem Maler die Waage verkauft.
4. Jetzt ist sie zu Hause und entspannt sich bei schöner Musik.

ACCESSIBLE-NEW:

1. Anna hatte einen erfolgreichen Tag.
2. Am Mittag sind einige Handwerker von der Baustelle nebenan in ihr Geschäft gekommen.
3. Unter anderem hat sie dem Maler eine Waage verkauft.
4. Jetzt ist sie zu Hause und entspannt sich bei schöner Musik.

ACCESSIBLE-ACCESSIBLE:

1. Anna hatte einen erfolgreichen Tag.
2. Am Mittag sind einige Handwerker von der Baustelle nebenan in ihr Geschäft gekommen, um sich die Restbestände bei den Küchengeräten anzusehen.
3. Unter anderem hat sie dem Maler die Waage verkauft.
4. Jetzt ist sie zu Hause und entspannt sich bei schöner Musik.

Story 6 (Kellner *server* – Vase *vase*)

NEW-NEW:

1. Claudia hatte einen erfolgreichen Tag.
2. Sie hat die Kunden auf dem Flohmarkt für viele ihrer Waren begeistern können.
3. Unter anderem hat sie einem Kellner eine Vase verkauft.
4. Jetzt geht sie nach Hause und legt sich sofort hin.

NEW-ACCESSIBLE:

1. Claudia hatte einen erfolgreichen Tag.
2. Sie hat die Kunden in ihrem Blumenladen für die restlichen Keramikartikel begeistern können.
3. Unter anderem hat sie einem Kellner die Vase verkauft.
4. Jetzt geht sie nach Hause und legt sich sofort hin.

ACCESSIBLE-NEW:

1. Claudia hatte einen erfolgreichen Tag im Geschäft.
2. Sie hat einige Kollegen vom Restaurant nebenan für viele ihrer Waren begeistern können.
3. Unter anderem hat sie dem Kellner eine Vase verkauft.
4. Jetzt geht sie nach Hause und legt sich sofort hin.

ACCESSIBLE-ACCESSIBLE:

1. Claudia hatte einen erfolgreichen Tag.
2. Sie hat einige Kollegen vom Restaurant nebenan für die restlichen Keramikartikel in ihrem Blumenladen begeistern können.
3. Unter anderem hat sie dem Kellner die Vase verkauft.
4. Jetzt geht sie nach Hause und legt sich sofort hin.

Story 7 (Oma *grandma* – Mantel *coat*)

NEW-NEW:

1. Rudi hatte einen erfolgreichen Tag.
2. An seinem Stand auf dem Flohmarkt sind viele Leute vorbeigekommen.
3. Unter anderem hat er einer Oma einen Mantel verkauft.
4. Jetzt entspannt er sich in der Badewanne.

NEW-ACCESSIBLE:

1. Rudi hatte einen erfolgreichen Tag.
2. Viele Kunden haben sich in seiner Boutique für die Einzelstücke der Herbst-Kollektion interessiert.
3. Unter anderem hat er einer Oma den Mantel verkauft.
4. Jetzt entspannt er sich in der Badewanne.

ACCESSIBLE-NEW:

1. Rudi hatte einen erfolgreichen Tag.
2. Unter den Kunden an seinem Stand auf dem Flohmarkt war auch eine Großfamilie.
3. Unter anderem hat er der Oma einen Mantel verkauft.
4. Jetzt entspannt er sich in der Badewanne.

ACCESSIBLE-ACCESSIBLE:

1. Rudi hatte einen erfolgreichen Tag.
2. Unter den Kunden in seiner Boutique war auch eine Großfamilie, die sich für die Einzelstücke der Herbst-Kollektion interessiert hat.
3. Unter anderem hat er der Oma den Mantel verkauft.
4. Jetzt entspannt er sich in der Badewanne.

Story 8 (Sänger *singer* – Kanne *can*)

NEW-NEW:

1. Sandra hatte einen erfolgreichen Tag.
2. An ihrem Stand auf dem Flohmarkt sind viele Leute vorbeigekommen.
3. Unter anderem hat sie einem Sänger eine Kanne verkauft.
4. Jetzt kocht sie sich Nudeln zum Abendessen.

NEW-ACCESSIBLE:

1. Sandra hatte einen erfolgreichen Tag in ihrem Teeladen.
2. Einige Kunden haben sich für die Einzelstücke der Porzellankollektion aus dem Vorjahr interessiert.
3. Unter anderem hat sie einem Sänger die Kanne verkauft.
4. Jetzt kocht sie sich Nudeln zum Abendessen.

ACCESSIBLE-NEW:

1. Sandra hatte einen erfolgreichen Tag auf dem Flohmarkt.
2. Es war viel los und wie immer gab es auch ein Live-Konzert.
3. Unter anderem hat sie dem Sänger eine Kanne verkauft.
4. Jetzt kocht sie sich Nudeln zum Abendessen.

ACCESSIBLE-ACCESSIBLE:

1. Sandra hatte einen erfolgreichen Tag auf dem Flohmarkt.
2. Wie immer gab es auch ein Live-Konzert und einige Leute haben sich für das alte Teeservice ihrer Oma interessiert.
3. Unter anderem hat sie dem Sänger die Kanne verkauft.
4. Jetzt kocht sie sich Nudeln zum Abendessen.

Practice stories

NEW-NEW Rabbi *rabbi* - Angel *fishing rod*:

1. Georg ist müde von der Arbeit.
2. Er hat den ganzen Tag im Fundbüro für Besucher Gegenstände herausgesucht.
3. Zum Schluss hat er einem Rabbi eine Angel gezeigt.
4. Jetzt geht er in seine Lieblingskneipe.

NEW-NEW Mutter *mother* - Lampe *lamp*:

1. Heute Morgen waren viele Kunden im Geschäft.
2. David war die ganze Zeit damit beschäftigt, Fragen zu beantworten.
3. Dann hat er einer Mutter eine Lampe verkauft.
4. Jetzt macht er Mittagspause.
